# Supplementary material for: Perceptions of risk and influences of choice in pregnant women with obesity. An evidence synthesis of qualitative research
Source: PLoS One. 2020 Jan 3;15(1):e0227325. doi: 10.1371/journal.pone.0227325 (PMC6941828; doi:10.1371/journal.pone.0227325)
Supplement: S2 Table — (DOCX) [file pone.0227325.s002.docx]

**S2 Table - Criteria used to define the components in the SPIDER literature search tool**

| SPIDER Element | Criteria |
| --- | --- |
| Sample | Search terms (free text and expanded synonyms of): “Obesity” OR “Pregnancy” OR “Maternal” OR “Antenatal” OR “Intrapartum” |
| Phenomenon of Interest | General and specific risks or choices of pregnancy determined data to be included at the full text review stage. |
| Design | Search terms (free text and expanded synonyms of): “Interview” OR “Ethnography” OR “Thematic Analysis” OR “Phenomenology” OR “Grounded Theory” etc. |
| Evaluation | Data on perceptions of risks and influences of choices determined papers to be included at the full text review stage. |
| Research Methods | Search terms (free text and expanded synonyms of): “Qualitative” |
