# Supplementary material for: Perceptions of risk and influences of choice in pregnant women with obesity. An evidence synthesis of qualitative research
Source: PLoS One. 2020 Jan 3;15(1):e0227325. doi: 10.1371/journal.pone.0227325 (PMC6941828; doi:10.1371/journal.pone.0227325)
Supplement: S3 Table — (DOCX) [file pone.0227325.s003.docx]

**S3 Table – Full search strategy and terms**

| **BNI** | **CINAHL** | **EMBASE** | **Medline** |
| --- | --- | --- | --- |
| ((exp OBESITY/ OR (obes*).ti,ab)  **AND**  (exp PREGNANCY/ OR exp "MATERNITY SERVICES"/ OR exp MIDWIFERY/ OR exp "MATERNITY SERVICES"/ OR exp CHILDBIRTH/ OR exp "ANTENATAL CARE"/ OR exp MIDWIFERY/ OR exp OBSTETRICS/ OR exp PREGNANCY/ OR (pregnan*).ti,ab OR (matern*).ti,ab))  **AND**  (exp "INTERVIEWS AND INTERVIEWING"/ OR (qualitative).ti,ab OR (thematic analysis).ti,ab OR (framework analysis).ti,ab OR (grounded theory).ti,ab OR (ethnograph*).ti,ab OR (phenomenolog*).ti,ab OR (interview).ti,ab) | (((obesity).ti,ab OR (obese).ti,ab OR exp "OBESITY, MORBID"/ OR exp OBESITY/)  **AND**  ((pregnan*).ti,ab OR (matern*).ti,ab OR exp PREGNANCY/ OR exp CHILDBIRTH/ OR exp LABOR/ OR exp "ATTITUDE TO PREGNANCY"/ OR exp "ATTITUDE OF HEALTH PERSONNEL"/ OR exp "OBSTETRIC PATIENTS"/ OR exp "MATERNAL HEALTH SERVICES"/ OR exp "PRENATAL CARE"/)) **AND**  ((qualitative).ti,ab OR (interview).ti,ab OR (framework analysis).ti,ab OR (thematic analysis).ti,ab OR (phenomenol* analysis).ti,ab OR (ethnograph*).ti,ab OR exp "QUALITATIVE STUDIES"/ OR exp "ETHNOGRAPHIC RESEARCH"/ OR exp "ETHNOLOGICAL RESEARCH"/ OR exp "ETHNONURSING RESEARCH"/ OR exp "GROUNDED THEORY"/ OR exp "PHENOMENOLOGICAL RESEARCH"/ OR exp "NATURALISTIC INQUIRY"/ OR exp "THEMATIC ANALYSIS"/) | ((exp OBESITY/ OR (obes*).ti,ab)  **AND**  ((pregnan*).ti,ab OR exp PREGNANCY/ OR exp "ATTITUDE TO PREGNANCY"/ OR exp "PREGNANT WOMAN"/ OR (matern*).ti,ab OR exp "MATERNAL HEALTH SERVICE"/ OR exp "MATERNAL OBESITY"/))  **AND**  (exp "QUALITATIVE RESEARCH"/ OR exp "QUALITATIVE ANALYSIS"/ OR exp "QUALITATIVE STUDIES"/ OR exp "QUALITATIVE STUDY"/ OR (qualitative).ti,ab OR (thematic analysis).ti,ab OR exp "THEMATIC ANALYSIS"/ OR exp "PSYCHOLOGICAL ASPECT"/ OR exp INTERVIEW/ OR exp "SEMI STRUCTURED INTERVIEW"/ OR exp "TELEPHONE INTERVIEW"/ OR exp "UNSTRUCTURED INTERVIEW"/ OR exp "INTERVIEW GUIDE"/ OR (interview*).ti,ab OR (framework analysis).ti,ab OR exp ETHNOGRAPHY/ OR (ethnograph*).ti,ab OR exp PHENOMENOLOGY/ OR (phenomenolog*).ti,ab OR exp "GROUNDED THEORY"/ OR (grounded theory).ti,ab) | ((exp OBESITY/ OR exp "OBESITY, MORBID"/ OR (obes*).ti,ab)  **AND**  (exp "PREGNANCY COMPLICATIONS"/ OR (exp PREGNANCY/ OR exp "PREGNANCY, HIGH-RISK"/) OR exp "PREGNANCY OUTCOME"/ OR exp "MATERNAL HEALTH SERVICES"/ OR exp "PERINATAL CARE"/ OR exp "PRENATAL CARE"/ OR (matern*).ti,ab OR (pregnan*).ti,ab))  **AND**  (exp "QUALITATIVE RESEARCH"/ OR (qualitative research).ti,ab OR exp ETHNOPSYCHOLOGY/ OR exp "QUALITATIVE RESEARCH"/ OR exp "GROUNDED THEORY"/ OR (interview).ti,ab OR (thematic analysis).ti,ab OR (framework analysis).ti,ab OR (grounded theory).ti,ab OR (ethnograph*).ti,ab OR (phenomenonolog*).ti,ab) |

| **Web of Science** | **Cochrane** |
| --- | --- |
| (TS=(obes*))OR(TI=(obes*))  **AND**  (TI=(pregnan* OR mater* OR midwife* OR midwive* OR *birth OR antenat* OR obstetr* OR labour OR labor OR prenatal OR gestation*)) OR (TS=(pregnan* OR mater* OR midwife* OR midwive* OR *birth OR antenat* OR obstetr* OR labour OR labor OR prenatal OR gestation*))  **AND**  (TI=(qualitat* OR interview* OR thematic OR framework OR ethnograph* OR phenomenology*)) OR (TS=(qualitat* OR interview* OR thematic OR framework OR ethnograph* OR phenomenology*)) | ((exp OBESITY/ OR (obes*)  **AND**  (exp PREGNANCY/ OR exp “MATERNAL HEALTH”/ OR (pregnan*) OR (gestation*) OR (matern*))  **AND**  (exp “QUALITATIVE RESEARCH”/ OR exp RISK/ OR exp “CHOICE BEHAVIOR”/ OR exp INTERVIEW/ OR exp “ANTHROPOLOGY, CULTURAL“ OR exp “SOCIOLOGICAL FACTORS”/ OR (qualitat*) OR (ethnograp*) OR (grounded theory) OR (thematic) OR (phenom*) |
