# Supplementary material for: Perceptions of risk and influences of choice in pregnant women with obesity. An evidence synthesis of qualitative research
Source: PLoS One. 2020 Jan 3;15(1):e0227325. doi: 10.1371/journal.pone.0227325 (PMC6941828; doi:10.1371/journal.pone.0227325)
Supplement: S4 Table — (DOCX) [file pone.0227325.s004.docx]

**S4 Table - Elements of the data extraction framework**

| - Perceptions of antenatal risks - Perceptions of labour and birth risks - Influences of non-lifestyle antenatal choices - Influences of labour and birth choices |
| --- |
