# Supplementary material for: Perceptions of risk and influences of choice in pregnant women with obesity. An evidence synthesis of qualitative research
Source: PLoS One. 2020 Jan 3;15(1):e0227325. doi: 10.1371/journal.pone.0227325 (PMC6941828; doi:10.1371/journal.pone.0227325)
Supplement: S5 Table — (DOCX) [file pone.0227325.s005.docx]

**S5 Table – Papers included and excluded following full text review**

| Title | First author | Year | Included | Reason for exclusion |
| --- | --- | --- | --- | --- |
| "As soon as you've had the baby that's it..." a qualitative study of 24 postnatal women on their experience of maternal obesity care pathways (1). | Dinsdale, S | 2016 | Yes |  |
| "If she wants to eat…and eat and eat…fine! It's gonna feed the baby": Pregnant women and partners' perceptions and experiences of pregnancy with a BMI >40 kg/m2 (2). | Keely, A | 2017 | Yes |  |
| ‘I just want to be normal’ – A qualitative study of pregnant women's blogs who present themselves as overweight or obese (3). | Lingetun, L | 2017 | Yes |  |
| A qualitative exploration of the lived experiences of pregnant women with BMI ≥35 kg/m2(4). | Charnley M | 2016 | No | *Insufficient data for extraction in a conference abstract* |
| A qualitative study exploring pregnant women's weight-related attitudes and beliefs in UK: the BLOOM study(5). | Padmanabhan, U | 2015 | No | *No differentiation between obese and normal weight women* |
| A Qualitative Study of Factors Affecting Pregnancy Weight Gain in African American Women(6). | Goodrich, K | 2013 | No | *No differentiation between obese and overweight women* |
| A Qualitative Study of Motivators and Barriers to Healthy Eating in Pregnancy for Low-Income, Overweight, African-American Mothers(7). | Reyes, N | 2013 | No | *No differentiation between obese and overweight women* |
| A qualitative study of the experiences of women who are obese and pregnant in the UK(8). | Furber, C | 2011 | Yes |  |
| A Qualitative Study of the Maternity Care Experiences of Women With Obesity(9). | Mandel, D | 2016 | No | *Insufficient data for extraction in a conference abstract. Duplicate of another paper (DeJoy, 2016)* |
| A Qualitative Study of the Maternity Care Experiences of Women with Obesity: 'More than Just a Number on the Scale'(10). | DeJoy, S | 2016 | Yes |  |
| A study of the experiences and concerns of obese childbearing women(11). | Mills, A | 2011 | No | Conference abstract |
| An exploration of obese pregnant women's views of being referred by their midwife to a weight management service(12). | Patel, C | 2013 | No | *Short article with no relevant data* |
| Beliefs, Barriers, and Preferences of European Overweight Women to Adopt a Healthier Lifestyle in Pregnancy to Minimize Risk of Developing Gestational Diabetes Mellitus: An Explorative Study(13). | Jelsma, JG | 2016 | No | *No differentiation between obese and overweight women* |
| Bonny babies? motherhood and nurturing in the age of obesity(14). | Keenan, J | 2010 | Yes |  |
| Communication with health professionals: The views of pregnant women with a raised BMI(15). | Cunningham, J | 2018 | Yes |  |
| Conceptions of pregnancy health and motivations for healthful behavior change among women in American Samoa(16). | Kocher, EL | 2018 | No | *Does not specify inclusion of obese women* |
| Cultural beliefs of severely obese pregnant women and their partners regarding gestational weight gain(17). | Keely, A | 2016 | No | Conference abstract |
| Diet and physical activity in pregnancy: a study exploring women's beliefs and behaviours(18). | Chana, R | 2019 | No | *Does not specify inclusion of obese women* |
| Disorders affecting quality of life during pregnancy: A qualitative study(19). | Kazemi, F | 2017 | No | *Does not specify inclusion of obese women* |
| Eat Well Keep Active: Qualitative findings from a feasibility and acceptability study of a brief midwife led intervention to facilitate healthful dietary and physical activity behaviours in pregnant women(20). | Warren, L | 2017 | No | *Women with BMI > 30kg/m2 excluded* |
| Emotional Experiences of Obese Women with Adequate Weight Variation During Pregnancy: a Qualitative Study(21). | Turato, ER | 2015 | Yes |  |
| Enablers of and barriers to making healthy change during pregnancy in overweight and obese women(22). | Sui, Z | 2013 | Yes |  |
| Enablers and barriers to physical activity in overweight and obese pregnant women : an analysis informed by the theoretical domains framework and COM-B model(23). | Flannery, C | 2018 | No | *No differentiation between obese and overweight women* |
| Excess Gestational Weight Gain in Low-Income Overweight and Obese Women: A Qualitative Study(24). | Anderson, C | 2015 | No | *No differentiation between obese and overweight women* |
| Experiences of a lifestyle intervention in obese pregnant women -- A qualitative study(25). | Fieril, KP | 2017 | Yes |  |
| Experiences related to health promotion behaviors in overweight pregnant women: a qualitative study(26). | Kazemi, AF | 2018 | No | *Does not specify inclusion of obese women* |
| Factors influencing the adoption of health promoting behaviors in overweight pregnant women: A qualitative study(27). | Fathnezhad-Kazemi, A | 2019 | No | *Does not specify inclusion of obese women* |
| Feelings of women who experienced a high-risk pregnancy: a descriptive study(28). | Wilhelm, LQ | 2015 | No | *Does not specify inclusion of obese women* |
| Get alongside us', women's experiences of being overweight and pregnant in Sydney, Australia(29). | Mills, A | 2013 | Yes |  |
| Health Behavior Change in Pregnant Women With Obesity(30). | McCloud, MB | 2018 | No | *Review article - not primary research* |
| Health beliefs and stages of changes to improve behaviors among obese and overweight women undergoing preconception care(31). | Malverdy, Z | 2016 | No | *Quantitative analyses only* |
| I Am Pregnant and Want to Do Better But I Can't: Focus Groups with Low-Income Overweight and Obese Pregnant Women(32). | Chang, M | 2015 | No | *No differentiation between obese and overweight women* |
| Inconsistent Weight Communication Among Prenatal Healthcare Providers and Patients: A Narrative Review(33). | Weeks, A | 2018 | No | *Review article - not primary research* |
| Influences on the food choices and physical activity behaviours of overweight and obese pregnant women: A qualitative study(34). | O'Brien, OA | 2017 | No | *No differentiation between obese and overweight women* |
| Knowledge, Attitudes and Provider Advice by Pre-Pregnancy Weight Status: A Qualitative Study of Pregnant Latinas With Excessive Gestational Weight Gain(35). | Wang, ML | 2015 | Yes |  |
| Lived experiences of routine antenatal dietetic services among women with obesity: A qualitative phenomenological study(36). | Heslehurst, N | 2017 | Yes |  |
| Lived experiences of women with co-existing BMI≥30 and Gestational Diabetes Mellitus(37). | Jarvie, R | 2017 | Yes |  |
| Maternal obesity in pregnancy: Women's understanding of risks(38). | Keely, A | 2011 | Yes |  |
| Maternal obesity support services: A qualitative study of the perspectives of women and midwives(39). | Furness PJ | 2011 | Yes |  |
| Maternal obesity; what is the best way to offer healthy lifestyle advice to women(40). | Smith, DM | 2012 | No | *Insufficient data for extraction in a conference abstract* |
| Maximising women's engagement with maternal obesity intervention: A qualitative study of pregnant women's experiences and priorities(41). | Heslehurst N | 2012 | No | *Duplicate* |
| Obese and nonobese postpartum women: complications, body image, and perceptions of the intrapartal experience(42). | Morin, KH | 1995 | No | *No data on perception of risks or influences of choices* |
| Obese mothers and their needs in antepartum care and mode of birth(43). | Schuecking, BA | 2010 | No | Conference abstract |
| Obese pregnant women with gestational diabetes mellitus: Fears, anxieties and beliefs as to becoming ill and being hospitalized(44). | Fregonese, A | 2009 | No | *Insufficient relevant data for extraction in a conference abstract* |
| Obese pregnant women's experience of their encounter with health professionals. Motivational interviewing as a tool to help health professionals communicate with obese pregnant women(45). | Lindhardt, C | 2012 | No | *Insufficient data for extraction in a conference abstract* |
| Obese women's experiences of encounters with midwives and physicians during pregnancy and childbirth(46). | Nyman, V | 2010 | Yes |  |
| Obese women's reasons for not attending a weight management service during pregnancy(47). | Olander, EK | 2013 | No | *Women interviewed at nine months postpartum* |
| Obesity and gestation: Emotional aspects involved(48). | Fregonese, A | 2009 | No | *Quantitative analyses only* |
| Obesity in Pregnancy: A Qualitative Approach to Inform an Intervention for Patients and Providers(49). | Kominiarek, M | 2015 | Yes |  |
| Obesity in pregnancy patient-reported outcomes: A qualitative study(50). | Dadouch, R | 2019 | No | *Doesn't study perception of risk or influence of choices* |
| Obesity: naming, blaming and shaming(51). | Wickham, S | 2009 | No | *Review article - not primary research* |
| Overweight and Obese Women’s Perceptions About Making Healthy Change During Pregnancy: A Mixed Method Study(52). | Sui, Z | 2013 | Yes |  |
| Patients' and professionals' experiences and perspectives of obesity in health-care settings: a synthesis of current research(53). | Mold, F | 2013 | No | *Does not specify inclusion of obese women* |
| Perception of overweight and obesity from different angles: A qualitative study(54). | Sikorski, C | 2012 | No | *Does not specify inclusion of obese women* |
| Physical activity in pregnancy: A qualitative study of the beliefs of overweight and obese pregnant women(55). | Weir, Z | 2010 | Yes |  |
| Physical activity in pregnant women with Class III obesity: A qualitative exploration of attitudes and behaviours(56). | Denison, FC | 2015 | Yes |  |
| Pregnancy and obesity: The experiences of obese women receiving antenatal care(57). | Williams, LT | 2015 | No | *Insufficient data for extraction in a conference abstract* |
| Pregnant women's knowledge of weight, weight gain, complications of obesity and weight management strategies in pregnancy(58), | Shub, A | 2013 | No | *No differentiation between obese and normal weight/overweight women* |
| Reasons for Late-Night Eating and Willingness to Change: A Qualitative Study in Pregnant Black Women(59). | Kroeger, EN | 2019 | No | *No differentiation between obese and overweight women* |
| Reports of obese women who had adequate weight gain during pregnancy - Qualitative research in southeastern Brazil(60). | Faria, D | 2013 | Yes |  |
| Reproducing stigma: Interpreting "overweight" and "obese" women's experiences of weight-based discrimination in reproductive healthcare(61). | Bombak, AE | 2016 | No | *No differentiation between obese and overweight women* |
| Seeing it through their eyes: a qualitative study of the pregnancy experiences of women with a body mass index of 30 or more(62). | Lavender, T | 2016 | Yes |  |
| Self-monitoring Lifestyle Behavior in Overweight and Obese Pregnant Women: Qualitative Findings(63). | Shieh, C | 2018 | No | *No differentiation between obese and overweight women* |
| Sterilization: decision-making by obese pregnant women(64). | Hastings-Tolsma, M | 2008 | Yes |  |
| The antenatal anaesthetic interview assists maternal decision making and does not increase anxiety in obese parturients(65). | Eley, VA | 2013 | No | *Insufficient data for extraction in a conference abstract. Duplicate of another paper (Eley, 2014).* |
| The comparison of outcomes reported by healthcare professionals and patients on the management of obesity in pregnancy(66). | Rosen, C | 2019 | No | *Reports on outcomes which are important to women not on perceptions of risk and influences of choice* |
| The effect of antenatal anaesthetic consultation on maternal decision-making, anxiety level and risk perception in obese pregnant women(67). | Eley, VA | 2014 | No | *Quantitative analyses only* |
| The experience of pregnant women with a body mass index >30 kg/m2 of their encounters with healthcare professionals(68). | Lindhardt, CL | 2013 | Yes |  |
| The perspectives of obese women receiving antenatal care: A qualitative study of women's experiences(69). | Knight-Agarwal, CR | 2016 | Yes |  |
| The views of women of above average weight about appropriate weight gain in pregnancy(70). | Wiles, R | 1998 | No | *Does not specify inclusion of obese women* |
| Unconscious collusion: An interpretative phenomenological analysis of the maternity care experiences of women with obesity (BMI≥30 kg/m²)(71). | Atkinson, S | 2017 | Yes |  |
| Using service-users' views to design a maternal obesity intervention(72). | Khazaezadeh, N | 2011 | Yes |  |
| Weight management during pregnancy: A qualitative study of women's and care providers' experiences and perspectives(73). | Holton, S | 2017 | Yes |  |
| Weight management during pregnancy: a qualitative thematic analysis on knowledge, perceptions and experiences of overweight and obese women in Singapore(74). | Loh, AZH | 2018 | No | *No differentiation between obese and overweight women* |
| Weight management guides for pregnant women with a body mass index (BMI) ≥ 40kg/m2: A qualitative exploration of their use in maternity care(75). | Smith, DM | 2013 | No | *Outlines women's views regarding physical weight management guides, not perceptions of risk and influences of choices antenatal or in labour.* |
| Weight-related risk perception among healthy and overweight pregnant women: a cross-sectional study(76). | de Jersey, SJ | 2015 | No | *Quantitative analyses only* |
| Weighty matters: Negotiating ‘fatness’ and ‘in-betweenness’ in early pregnancy(77). | Nash, M | 2012 | No | *No differentiation between obese and overweight women* |
| What women perceive as reasons for their unhealthy weight gain during and after pregnancy: A qualitative interview study(78). | Christenson, A | 2016 | No | *Does not specify inclusion of obese women* |
| When will it show? exploration of the pregnancy experience of women with a body mass index over 40 kg/m2 in preconception(79). | Gilbert, L | 2013 | No | *Insufficient data for extraction in a conference abstract* |
| Why don’t many obese pregnant and post-natal women engage with a weight management service?(80) | Atkinson, L | 2013 | No | *Evaluates a service which was offered up until two years postnatally* |
| Women with a BMI ≥ 30 kg/m² and their experience of maternity care: A meta ethnographic synthesis(81). | Jones, C | 2017 | No | *Review article - not primary research* |
| Women's experiences of changes in eating during pregnancy: A qualitative study in Dunedin, New Zealand(82). | Paterson, H | 2016 | No | *No differentiation between obese and overweight women* |
| Women's perspectives are required to inform the development of maternal obesity services: a qualitative study of obese pregnant women's experiences(83). | Heslehurst, N | 2015 | Yes |  |

**References**

1. Dinsdale S, Branch K, Cook L, Shucksmith J. "As soon as you've had the baby that's it..." a qualitative study of 24 postnatal women on their experience of maternal obesity care pathways. BMC Public Health. 2016;16:625.

2. Keely A, Cunningham-Burley S, Elliott L, Sandall J, Whittaker A. "If she wants to eat...and eat and eat...fine! It's gonna feed the baby": Pregnant women and partners' perceptions and experiences of pregnancy with a BMI >40kg/m(2). Midwifery. 2017;49:87-94.

3. Lingetun L, Fungbrant M, Claesson IM, Baggens C. 'I just want to be normal' - A qualitative study of pregnant women's blogs who present themselves as overweight or obese. Midwifery. 2017;49:65-71.

4. Charnley MS, Coufopoulos A, Weeks A, Abayomi JC. A qualitative exploration of the lived experiences of pregnant women with BMI ⩾35 kg/m2. P Nutr Soc. 2016;75(OCE3).

5. Luni Y, Borakati A, Matah A, Skeats K, Eedarapalli P. A prospective cohort study evaluating the cost-effectiveness of carbetocin for prevention of postpartum haemorrhage in caesarean sections. J Obstet Gynaecol. 2017;37(5):601-4.

6. Goodrich K, Cregger M, Wilcox S, Liu J. A qualitative study of factors affecting pregnancy weight gain in African American women. Matern Child Health J. 2013;17(3):432-40.

7. Reyes NR, Klotz AA, Herring SJ. A qualitative study of motivators and barriers to healthy eating in pregnancy for low-income, overweight, African-American mothers. J Acad Nutr Diet. 2013;113(9):1175-81.

8. Furber CM, McGowan L. A qualitative study of the experiences of women who are obese and pregnant in the UK. Midwifery. 2011;27(4):437-44.

9. Mandel D, DeJoy SB. A Qualitative Study of the Maternity Care Experiences of Women With Obesity. Journal of Obstetric, Gynecologic & Neonatal Nursing. 2016;45(3):S48.

10. DeJoy SB, Bittner K, Mandel D. A Qualitative Study of the Maternity Care Experiences of Women with Obesity: "More than Just a Number on the Scale". J Midwifery Womens Health. 2016;61(2):217-23.

11. Mills A, Dahlen H, Schmied V. A study of the experiences and concerns of obese childbearing women. Journal of Paediatrics and Child Health. 2011;47((Suppl. 1)):8-59.

12. Patel C, Atkinson L, Olander EK. An exploration of obese pregnant women's views of being referred by their midwife to a weight management service. Sex Reprod Healthc. 2013;4(4):139-40.

13. Jelsma JG, van Leeuwen KM, Oostdam N, Bunn C, Simmons D, Desoye G, et al. Beliefs, Barriers, and Preferences of European Overweight Women to Adopt a Healthier Lifestyle in Pregnancy to Minimize Risk of Developing Gestational Diabetes Mellitus: An Explorative Study. J Pregnancy. 2016;2016:3435791.

14. Keenan J, Stapleton H. Bonny babies? Motherhood and nurturing in the age of obesity. Health, Risk & Society. 2010;12(4):369-83.

15. Cunningham J, Endacott R, Gibbons D. Communication with health professionals: The views of pregnant women with a raised BMI. British Journal of Midwifery. 2018;26(9):598-604.

16. Kocher EL, Sternberg Lamb JM, McGarvey ST, Faiai Mu, Muasau-Howard BT, Hawley NL. Conceptions of pregnancy health and motivations for healthful behavior change among women in American Samoa. Women and Birth. 2018;31(1):e32-e41.

17. Keely A, Elliott L, Cunningham-Burley S. Cultural beliefs of severely obese pregnant women and their partners regarding gestational weight gain. British Journal of Obstetrics and Gynaecology. 2016;123(S1):54-5.

18. Chana R, Haith-Cooper M. Diet and physical activity in pregnancy: a study exploring women's beliefs and behaviours. British Journal of Midwifery. 2019;27(5):297-304.

19. Kazemi F, Nahidi F, Kariman N. Disorders Affecting Quality of Life During Pregnancy: A Qualitative Study. J Clin Diagn Res. 2017;11(4):QC06-QC10.

20. Warren L, Rance J, Hunter B. Eat Well Keep Active: Qualitative findings from a feasibility and acceptability study of a brief midwife led intervention to facilitate healthful dietary and physical activity behaviours in pregnant women. Midwifery. 2017;49:117-23.

21. Turato ER, Faria-Schutzer DB, Surita FGC, Vieira CM. Emotional Experiences of Obese Women with Adequate Weight Variation During Pregnancy: a Qualitative Study. Eur Psychiatry. 2015;30.

22. Sui Z, Turnbull D, Dodd J. Enablers of and barriers to making healthy change during pregnancy in overweight and obese women. Australas Med J. 2013;6(11):565-77.

23. Flannery C, McHugh S, Anaba AE, Clifford E, O'Riordan M, Kenny LC, et al. Enablers and barriers to physical activity in overweight and obese pregnant women: an analysis informed by the theoretical domains framework and COM-B model. BMC Pregnancy Childbirth. 2018;18(1):178.

24. Anderson CK, Walch TJ, Lindberg SM, Smith AM, Lindheim SR, Whigham LD. Excess Gestational Weight Gain in Low-Income Overweight and Obese Women: A Qualitative Study. J Nutr Educ Behav. 2015;47(5):404-11 e1.

25. Fieril DP, Olsen PF, Glantz D, Premberg DA. Experiences of a lifestyle intervention in obese pregnant women - A qualitative study. Midwifery. 2017;44:1-6.

26. Kazemi AF, Hajian S. Experiences related to health promotion behaviors in overweight pregnant women: a qualitative study. Reproductive Health. 2018;15.

27. Fathnezhad-Kazemi A, Hajian S. Factors influencing the adoption of health promoting behaviors in overweight pregnant women: a qualitative study. BMC Pregnancy Childbirth. 2019;19(1):43.

28. Wilhelm LA, Alves CN, Demori CC, Silva SCd, Meincke SMK, Ressel LB. Feelings of women who experienced a high-risk pregnancy: a descriptive study. 2015. 2015;14(3):10.

29. Mills A, Schmied VA, Dahlen HG. 'Get alongside us', women's experiences of being overweight and pregnant in Sydney, Australia. Matern Child Nutr. 2013;9(3):309-21.

30. McCloud MB. Health Behavior Change in Pregnant Women With Obesity. Nurs Womens Health. 2018;22(6):471-80.

31. Malverdy Z, Kazemi A. Health beliefs and stages of changes to improve behaviors among obese and overweight women undergoing preconception care. Iranian journal of nursing and midwifery research. 2016;21(6):595-600.

32. Chang MW, Nitzke S, Buist D, Cain D, Horning S, Eghtedary K. I am pregnant and want to do better but i can't: focus groups with low-income overweight and obese pregnant women. Matern Child Health J. 2015;19(5):1060-70.

33. Weeks A, Liu RH, Ferraro ZM, Deonandan R, Adamo KB. Inconsistent Weight Communication Among Prenatal Healthcare Providers and Patients: A Narrative Review. Obstet Gynecol Surv. 2018;73(8):423-32.

34. O'Brien OA, Lindsay KL, McCarthy M, McGloin AF, Kennelly M, Scully HA, et al. Influences on the food choices and physical activity behaviours of overweight and obese pregnant women: A qualitative study. Midwifery. 2017;47:28-35.

35. Wang ML, Arroyo J, Druker S, Sankey HZ, Rosal MC. Knowledge, Attitudes and Provider Advice by Pre-Pregnancy Weight Status: A Qualitative Study of Pregnant Latinas With Excessive Gestational Weight Gain. Women Health. 2015;55(7):805-28.

36. Heslehurst N, Dinsdale S, Brandon H, Johnston C, Summerbell C, Rankin J. Lived experiences of routine antenatal dietetic services among women with obesity: A qualitative phenomenological study. Midwifery. 2017;49:47-53.

37. Jarvie R. Lived experiences of women with co-existing BMI >= 30 and Gestational Diabetes Mellitus. Midwifery. 2017;49:79-86.

38. Keely A, Gunning M, Denison F. Maternal obesity in pregnancy: Women’s understanding of risks. British Journal of Midwifery. 2011;19(6):364-9.

39. Furness PJ, McSeveny K, Arden MA, Garland C, Dearden AM, Soltani H. Maternal obesity support services: a qualitative study of the perspectives of women and midwives. BMC Pregnancy Childbirth. 2011;11:69.

40. Smith DM, Whitworth M, Sibley C, Roberts S, Taylor W, Lavender T. M240 Maternal Obesity; What Is the Best Way to Offer Healthy Lifestyle Advice to Women. International Journal of Gynecology & Obstetrics. 2012;119:S610-S.

41. Heslehurst N, Russell SL, Johnston C, Brandon H, Barber A, Rankin J, et al. Maximising women's engagement with maternal obesity intervention: A qualitative study of pregnant women's experiences and priorities. Obesity Facts. 2012;5(s1):234.

42. Morin KH. Obese and nonobese postpartum women: complications, body image, and perceptions of the intrapartal experience. Appl Nurs Res. 1995;8(2):81-7.

43. Schuecking B, Makowsky K, von Moeller K. Obese mothers and their needs in antepartum care and mode of birth. Arch Gynecol Obstet. 2010;282(Suppl 1):S3.

44. Fregonese A, Theodoro L, Pinto L, Malheiros C, Hsu L, Bruscato W. Obese Pregnant Women with Gestational Diabetes Mellitus: Fears, Anxieties and Beliefs as to Becoming Ill and Being Hospitalized. Obesity Surgery. 2009;19:1031.

45. Lindhardt C, Hansen H, Rubak S, Mogensen O, Joergensen J. Obese pregnant women's experience of their encounter with health profesionals. Motivational interviewing as a tool to help health professionals communicate with obese pregnant women. Acta Obstet Gynecol Scand. 2012;91:105-6.

46. Nyman VM, Prebensen AK, Flensner GE. Obese women's experiences of encounters with midwives and physicians during pregnancy and childbirth. Midwifery. 2010;26(4):424-9.

47. Olander EK, Atkinson L. Obese women's reasons for not attending a weight management service during pregnancy. Acta Obstet Gynecol Scand. 2013;92(10):1227-30.

48. Fregonese A, Pinto L, Theodoro L, Hsu L, Bruscato W, Malheiros C. Obesity and Gestation: Emotional Aspects Involved. Obesity Surgery. 2009;19:1033.

49. Kominiarek MA, Gay F, Peacock N. Obesity in Pregnancy: A Qualitative Approach to Inform an Intervention for Patients and Providers. Matern Child Health J. 2015;19(8):1698-712.

50. Dadouch R, Hall C, D'Souzoa RD. Obesity in Pregnancy Patient-Reported Outcomes: A Qualitative Meta-Synthesis [40N]. Obstetrics & Gynecology. 2019;133:161S.

51. Wickham S. Obesity: naming, blaming and shaming. Pract Midwife. 2009;12(10):20-1.

52. Sui Z, Turnbull DA, Dodd JM. Overweight and obese women's perceptions about making healthy change during pregnancy: a mixed method study. Matern Child Health J. 2013;17(10):1879-87.

53. Mold F, Forbes A. Patients' and professionals' experiences and perspectives of obesity in health-care settings: a synthesis of current research. Health Expect. 2013;16(2):119-42.

54. Sikorski C, Riedel C, Luppa M, Schulze B, Werner P, Konig HH, et al. Perception of overweight and obesity from different angles: a qualitative study. Scand J Public Health. 2012;40(3):271-7.

55. Weir Z, Bush J, Robson SC, McParlin C, Rankin J, Bell R. Physical activity in pregnancy: a qualitative study of the beliefs of overweight and obese pregnant women. BMC Pregnancy Childbirth. 2010;10:18.

56. Denison FC, Weir Z, Carver H, Norman JE, Reynolds RM. Physical activity in pregnant women with Class III obesity: A qualitative exploration of attitudes and behaviours. Midwifery. 2015;31(12):1163-7.

57. Williams LT, Knight-Agarwal CR, Davis D, Davey R. Pregnancy and obesity: The experiences of obese women receiving antenatal care. Obesity Facts. 2015;8(s1):16.

58. Shub A, Huning EY, Campbell KJ, McCarthy EA. Pregnant women's knowledge of weight, weight gain, complications of obesity and weight management strategies in pregnancy. BMC Res Notes. 2013;6:278.

59. Kroeger EN, Carson TL, Baskin ML, Langaigne A, Schneider CR, Bertrand B, et al. Reasons for Late-Night Eating and Willingness to Change:A Qualitative Study in Pregnant Black Women. Journal of Nutrition Education and Behavior. 2019;51(5):598-607.

60. Faria D, Vieira C, Surita FGC, Turato ER. Reports of obese women who had adequate weight gain during pregnancy - qualitative research in southeastern Brazil. Psychother Psychosom. 2013;82:29-.

61. Bombak AE, McPhail D, Ward P. Reproducing stigma: Interpreting "overweight" and "obese" women's experiences of weight-based discrimination in reproductive healthcare. Soc Sci Med. 2016;166:94-101.

62. Lavender T, Smith DM. Seeing it through their eyes: a qualitative study of the pregnancy experiences of women with a body mass index of 30 or more. Health Expect. 2016;19(2):222-33.

63. Shieh C, Draucker CB. Self-monitoring Lifestyle Behavior in Overweight and Obese Pregnant Women Qualitative Findings. Clin Nurse Spec. 2018;32(2):81-9.

64. Hastings-Tolsma M, Clark L, Nodine P, Teal S. Sterilization decision making among medically at-risk obese pregnant women. Qual Health Res. 2010;20(6):743-54.

65. Eley VA, Donovan KJ, Walters E, Brijball R, Eley D. The antenatal anaesthetic interview assists maternal decision making and does not increase anxiety in obese parturients. International Journal of Obstetric Anesthesia. 2013;22:S15.

66. Rosen C, Dadouch R, Parsons J, D'Souza R. The comparison of outcomes reported by healthcare professionals and patients on the management of obesity in pregnancy. Journal of Evidence-Based Medicine. 2019;12(S1):9.

67. Eley VA, Donovan K, Walters E, Brijball R, Eley DS. The effect of antenatal anaesthetic consultation on maternal decision-making, anxiety level and risk perception in obese pregnant women. Int J Obstet Anesth. 2014;23(2):118-24.

68. Lindhardt CL, Rubak S, Mogensen O, Lamont RF, Joergensen JS. The experience of pregnant women with a body mass index >30 kg/m(2) of their encounters with healthcare professionals. Acta Obstet Gynecol Scand. 2013;92(9):1101-7.

69. Knight-Agarwal CR, Williams LT, Davis D, Davey R, Shepherd R, Downing A, et al. The perspectives of obese women receiving antenatal care: A qualitative study of women's experiences. Women Birth. 2016;29(2):189-95.

70. Wiles R. The views of women of above average weight about appropriate weight gain in pregnancy. Midwifery. 1998;14(4):254-60.

71. Atkinson S, McNamara PM. Unconscious collusion: An interpretative phenomenological analysis of the maternity care experiences of women with obesity (BMI>/=30kg/m(2)). Midwifery. 2017;49:54-64.

72. Khazaezadeh N, Pheasant H, Bewley S, Mohiddin A, Oteng-Ntim E. Using service-users’ views to design a maternal obesity intervention. British Journal of Midwifery. 2011;19(1):49-56.

73. Holton S, East C, Fisher J. Weight management during pregnancy: a qualitative study of women's and care providers' experiences and perspectives. BMC Pregnancy Childbirth. 2017;17(1):351.

74. Loh AZH, Oen KQX, Koo IJY, Ng YW, Yap JCH. Weight management during pregnancy: a qualitative thematic analysis on knowledge, perceptions and experiences of overweight and obese women in Singapore. Glob Health Action. 2018;11(1):1499199.

75. Smith DM, Ward C, Forbes S, Reynolds RM, Denison FC. Weight management guides for pregnant women with a body mass index (BMI) ≥ 40kg/m2: A qualitative exploration of their use in maternity care. Health Education Journal. 2012;72(2):216-21.

76. de Jersey SJ, Callaway LK, Daniels LA, Nicholson JM. Weight-related risk perception among healthy and overweight pregnant women: a cross-sectional study. Journal Of Perinatology. 2015;35:683.

77. Nash M. Weighty matters: Negotiating ‘fatness’ and ‘in-betweenness’ in early pregnancy. Feminism & Psychology. 2012;22(3):307-23.

78. Christenson A, Hemmingsson E, Reynisdottir S, Torgerson J, Johansson E. What women perceive as reasons for their unhealthy weight gain during and after pregnancy: A qualitative interview study. Obes Facts. 2016;9(S1):258.

79. Gilbert L. When Will it Show? Exploration of the Pregnancy Experience of Women With a Body Mass Index over 40 kg/m2 in Preconception. Canadian Journal of Diabetes. 2013;37:S267.

80. Atkinson L, Olander EK, French DP. Why don’t many obese pregnant and post-natal women engage with a weight management service? Journal of Reproductive and Infant Psychology. 2013;31(3):245-56.

81. Jones C, Jomeen J. Women with a BMI >/= 30kg/m(2) and their experience of maternity care: A meta ethnographic synthesis. Midwifery. 2017;53:87-95.

82. Paterson H, Hay-Smith, E.J.C., Treharne GJ. Women's experiences of changes in eating during pregnancy: A qualitative study in Dunedin, New Zealand. New Zealand College of Midwives Journal. 2016(52):5-11.

83. Heslehurst N, Russell S, Brandon H, Johnston C, Summerbell C, Rankin J. Women's perspectives are required to inform the development of maternal obesity services: a qualitative study of obese pregnant women's experiences. Health Expect. 2015;18(5):969-81.
