# Supplementary material for: Perceptions of risk and influences of choice in pregnant women with obesity. An evidence synthesis of qualitative research
Source: PLoS One. 2020 Jan 3;15(1):e0227325. doi: 10.1371/journal.pone.0227325 (PMC6941828; doi:10.1371/journal.pone.0227325)
Supplement: S6 Table — (DOCX) [file pone.0227325.s006.docx]

**S6 Table – Characteristics of Included Studies**

| **Author** | **Country** | **Study aim or objective** | **Source of participants** | **Inclusion criteria** | **Obese n=** | **Method of data collection** | **Method of analysis** | **Academic background of interviewer** |
| --- | --- | --- | --- | --- | --- | --- | --- | --- |
| Dinsdale, S (2016)(1) | UK | To explore and gain understanding of recent mother's general views and experiences of the maternal obesity care pathways offered in a single UK hospital. | Women on one of the three pathways | Women with BMI>30kg/m^2^ attending one of three maternal obesity care pathways in North East England. | 24 | Semi-structured interviews | Thematic content analysis | Not from a clinical background or linked to maternity services |
| Keely, A (2017)(2) | UK | To explore the experiences, attitudes and health-related behaviours of pregnant women with a BMI > 40 kg/m^2^; and to identify the factors and considerations which shape their beliefs, experiences and behaviours. | Antenatal clinic | Pregnant women with BMI>40kg/m^2^. | 11 | Semi-structured interviews | Thematic content analysis | Not described |
| Furber, C (2011)(3) | UK | To explore the experiences related to obesity in women with a body mass index BMI>35 kg/m^2^ during the childbearing process. | Maternity service in the north of England | Women with BMI>35kg/m^2^ who were older than 16 years of age. | 19 | Semi-structured interviews and field notes | Framework analysis method | Lecturer in Midwifery |
| DeJoy, SB (2016)(4) | USA | To understand the experiences of women with obesity in the US maternity care system. | Online communities | Pregnant and recently postpartum women in the United States who self-identified as 'plus size' and self-reported BMI>30kg/m^2^. | 16 | Telephone interview | Inductive analysis | Assistant professor in Health Department |
| Keenan, J (2010)(5) | UK | To explore how the medicalisation and moralisation of large bodies plays out in women's reported interactions with maternity professionals in pregnancy, birth and the months that follow. | Antenatal clinic, personal contacts and 'snow balling' | Women with BMI>30kg/m^2^ as calculated from self-reported height and weight. | 60 | Interview | Thematic analysis | Not described |
| Mills, A (2013)(6) | Australia | To explore the perceptions and experiences of overweight pregnant women attending two maternity units in Sydney, Australia. | Two hospitals | Women with a BMI>30kg/m^2^ who were in their third trimester of pregnancy or had recently given birth. | 14 | Qualitative descriptive method | Thematic analysis | Not described |
| Heslehurst, N (2017)(7) | UK | To understand the lived experiences and views of being referred to an antenatal dietetic service from the perspective of pregnant women with obesity. | Antenatal dietetics service through postal recruitment | Pregnant women with a BMI>30kg/m^2^ attending an obesity-specific antenatal dietetic service. | 15 | In depth unstructured interview | Thematic analysis | Lecturer in Public Health |
| Jarvie, R (2017)(8) | UK | To explore the lived experiences of women with co-existing maternal obesity (BMI≥30kg/m^2^) and gestational diabetes mellitus during pregnancy and the post-birth period (<3 months post-birth). | Diabetic antenatal clinic | Pregnant women with BMI>30kg/m^2^, with a diagnosis of gestational diabetes. | 27 | "Loosely structured" in depth interview | Thematic analysis | Not described |
| Keely, A (2011)(9) | UK | To explore obese women’s perceptions of obesity as a risk factor in pregnancy and their experiences of NHS maternity care. | Specialist obstetric anaesthetist clinic | Pregnant women with BMI >40kg/m^2^ who attended a specialist obstetric anaesthetist clinic at the Royal Infirmary in Edinburgh. | 8 | Open-ended, semi structured interview | Thematic analysis | Not described |
| Furness PJ (2011)(10) | UK | To explore the experiences and perceptions of pregnant women and midwives regarding existing support for weight management in pregnancy and their ideas for service development. | Community clinics and hospital | Maternity service uses with BMI>30kg/m^2^ in Doncaster, UK. | 6 | Semi-structured focus group | Thematic analysis | Not described |
| Nyman, VMK (2010)(11) | Sweden | To describe obese women’s experiences of encounters with midwives and physicians during pregnancy and childbirth. | Hospital in western Sweden | Women with BMI>30kg/m^2^ who had given birth at a hospital in Western Sweden. | 10 | Interview | Phenomenological method | Not described |
| Kominiarek, M (2015)(12) | USA | To investigate perceptions of minority pregnant women and providers about obesity and gestational weight gain, and to explore strategies to improve management of obesity in pregnancy with an emphasis on group prenatal care. | Hospital | Non-Hispanic black pregnant women with BMI>30kg/m^2^. | 16 | Semi-structured interview | Thematic analysis | Experienced focus group moderator |
| Sui, Z (2013)(13) | USA | To evaluate overweight and obese women’s perceptions of making behaviour change during pregnancy. | 3 public maternity hospital | Women with BMI>25kg/m^2^ and singleton pregnancies | 5 | Self-administered questionnaires and semi -structured interview | Deductive approach | Health science researcher |
| Denison, FC (2015) (14) | UK | To explore the barriers and facilitators to physical activity and lifestyle interventions in pregnant women with Class III obesity (body mass index >40 kg/m^2^). | Antenatal metabolic clinic | Pregnant women with BMI>40kg/m^2^. | 13 | Semi-structured, in-depth interviews | Framework approach | Research assistants |
| Lavender, T (2016)(15) | UK | To gain insight into the experience of pregnant women with BMI ≥ 30 kg/m^2^, when accessing maternity services and attending a community lifestyle programme | Lifestyle programme | Women with BMI>30kg/m^2^ who were attending a lifestyle programme at 4-6 weeks postpartum | 34 | Semi-structured interview | Thematic analysis | Qualified midwives |
| Hastings-Tolsma, M (2008)(16) | USA | To explore the components of sterilization decision making by pregnant obese women before and after their chosen sterilisation procedures and elicit the domains of the decision-making process used by women selecting permanent sterilization. | Those who have already consented for sterilization | Obese pregnant women who opt for a permanent sterilisation procedure during pregnancy. | 15 | Semi-structured interview | Grounded theory | Not described |
| Lindhardt, CL (2013)(17) | Denmark | To examine the experience of women with a pre-pregnant BMI >30 kg/m^2^, in their encounters with healthcare professionals during pregnancy | Midwife led antenatal clinic | Women with a pre-pregnant BMI of >30kg/m^2^. | 16 | Semi-structured in-depth interview | Phenomenological analysis | Not described |
| Knight-Agarwal, CR. (2016)(18) | Australia | To investigate the perspectives of pregnant women with a body mass index (BMI) of 30 kg/m^2^ receiving antenatal care | Hospital | Pregnant women with BMI>30kg/m^2^. | 16 | Semi-structured interview | Phenomenological analysis | Not described |
| Atkinson, S (2017)(19) | Ireland | To generate knowledge and provide healthcare professionals with essential information and understanding of a phenomenon, such as obesity in pregnancy. | Postnatal ward | Women with a BMI>30kg/m^2^ who had given birth to a liveborn term neonate (not admitted to NICU). | 15 | Semi- structured interview | Interpretative phenomenological analysis | Not described |
| Khazaezadeh, N (2011) (20) | UK | To identify and understand the health-care needs of obese pregnant women in Lambeth in south-east London. | Postnatal ward, fertility clinic | Recently pregnant women with BMI>30kg/m^2^. | 6 | Semi- structured interview | Framework analysis method | Not described |
| Holton, S (2017)(21) | Australia | To describe women’s and midwives’ experiences and perspectives of care for weight management during pregnancy in Melbourne, Australia | Hospital | English-speaking women aged >18 years, with or without a high BMI and receiving pregnancy care at the Monash Medical Centre, Australia. | 6 | Semi-structured interview | Thematic analysis | Not described |
| Heslehurst, N (2015)(22) | UK | To explore obese pregnant women’s experiences to better understand factors which need to be considered when developing services that women will find acceptable and utilise | Dietetic clinic | Obese women referred to an antenatal dietetic service in the Northeast of England. | 15 | Low structured depth interview | Thematic content analysis | Lecturer in Public Health |
| Cunningham, J (2018)(23) | UK | To explore the perceived impact of interactions with health professionals, and to investigate participants’ understanding of raised BMI and raised risk. | Hospital | Women with BMI 30kg/m2 or more and who were pregnant at the time of study recruitment. | 11 | 9 individual interviews and 1 group interview | Thematic analysis | Midwife researcher |

**References**

1. Dinsdale S, Branch K, Cook L, Shucksmith J. "As soon as you've had the baby that's it..." a qualitative study of 24 postnatal women on their experience of maternal obesity care pathways. BMC Public Health. 2016;16:625.

2. Keely A, Cunningham-Burley S, Elliott L, Sandall J, Whittaker A. "If she wants to eat...and eat and eat... fine! It's gonna feed the baby": Pregnant women and partners' perceptions and experiences of pregnancy with a BMI > 40 kg/m(2). Midwifery. 2017;49:87-94.

3. Furber CM, McGowan L. A qualitative study of the experiences of women who are obese and pregnant in the UK. Midwifery. 2011;27(4):437-44.

4. DeJoy SB, Bittner K, Mandel D. A Qualitative Study of the Maternity Care Experiences of Women with Obesity: "More than Just a Number on the Scale". Journal of Midwifery & Womens Health. 2016;61(2):217-23.

5. Keenan J, Stapleton H. Bonny babies? Motherhood and nurturing in the age of obesity. Health, Risk & Society. 2010;12(4):369-83.

6. Mills A, Schmied VA, Dahlen HG. 'Get alongside us', women's experiences of being overweight and pregnant in Sydney, Australia. Maternal and Child Nutrition. 2013;9(3):309-21.

7. Heslehurst N, Dinsdale S, Brandon H, Johnston C, Summerbell C, Rankin J. Lived experiences of routine antenatal dietetic services among women with obesity: A qualitative phenomenological study. Midwifery. 2017;49:47-53.

8. Jarvie R. Lived experiences of women with co-existing BMI>/=30 and Gestational Diabetes Mellitus. Midwifery. 2017;49:79-86.

9. Keely A, Gunning M, Denison F. Maternal obesity in pregnancy: Women’s understanding of risks. British Journal of Midwifery. 2011;19(6):364-9.

10. Furness PJ, McSeveny K, Arden MA, Garland C, Dearden AM, Soltani H. Maternal obesity support services: a qualitative study of the perspectives of women and midwives. Bmc Pregnancy and Childbirth. 2011;11.

11. Nyman VM, Prebensen AK, Flensner GE. Obese women's experiences of encounters with midwives and physicians during pregnancy and childbirth. Midwifery. 2010;26(4):424-9.

12. Kominiarek MA, Gay F, Peacock N. Obesity in Pregnancy: A Qualitative Approach to Inform an Intervention for Patients and Providers. Maternal and Child Health Journal. 2015;19(8):1698-712.

13. Sui Z, Turnbull DA, Dodd JM. Overweight and Obese Women's Perceptions About Making Healthy Change During Pregnancy: A Mixed Method Study. Maternal and Child Health Journal. 2013;17(10):1879-87.

14. Denison FC, Weir Z, Carver H, Norman JE, Reynolds RM. Physical activity in pregnant women with Class III obesity: A qualitative exploration of attitudes and behaviours. Midwifery. 2015;31(12):1163-7.

15. Lavender T, Smith DM. Seeing it through their eyes: a qualitative study of the pregnancy experiences of women with a body mass index of 30 or more. Health Expectations. 2016;19(2):222-33.

16. Hastings-Tolsma M, Clark L, Nodine P, Teal S. Sterilization Decision Making Among Medically At-Risk Obese Pregnant Women. Qualitative Health Research. 2010;20(6):743-54.

17. Lindhardt CL, Rubak S, Mogensen O, Lamont RF, Joergensen JS. The experience of pregnant women with a body mass index > 30 kg/m(2) of their encounters with healthcare professionals. Acta Obstetricia Et Gynecologica Scandinavica. 2013;92(9):1101-7.

18. Knight-Agarwal CR, Williams LT, Davis D, Davey R, Shepherd R, Downing A, et al. The perspectives of obese women receiving antenatal care: A qualitative study of women's experiences. Women and Birth. 2016;29(2):189-95.

19. Atkinson S, McNamara PM. Unconscious collusion: An interpretative phenomenological analysis of the maternity care experiences of women with obesity (BMI >= 30 kg/m(2)). Midwifery. 2017;49:54-64.

20. Khazaezadeh N, Pheasant H, Bewley S, Mohiddin A, Oteng-Ntim E. Using service-users’ views to design a maternal obesity intervention. British Journal of Midwifery. 2011;19(1):49-56.

21. Holton S, East C, Fisher J. Weight management during pregnancy: a qualitative study of women's and care providers' experiences and perspectives. BMC Pregnancy Childbirth. 2017;17(1):351.

22. Heslehurst N, Russell S, Brandon H, Johnston C, Summerbell C, Rankin J. Women's perspectives are required to inform the development of maternal obesity services: a qualitative study of obese pregnant women's experiences. Health Expectations. 2015;18(5):969-81.

23. Cunningham J, Endacott R, Gibbons D. Communication with health professionals: The views of pregnant women with a raised BMI. British Journal of Midwifery. 2018;26(9):598-604.
