# Supplementary material for: Perceptions of risk and influences of choice in pregnant women with obesity. An evidence synthesis of qualitative research
Source: PLoS One. 2020 Jan 3;15(1):e0227325. doi: 10.1371/journal.pone.0227325 (PMC6941828; doi:10.1371/journal.pone.0227325)
Supplement: S7 Table — (DOCX) [file pone.0227325.s007.docx]

**S7 Table – CASP Assessment of Included Studies**

|  | **Are the results of the study valid?** | | | | | | **What are the results?** | | | **Will the results help locally?** |
| --- | --- | --- | --- | --- | --- | --- | --- | --- | --- | --- |
|  | Was there a clear statement of the aims of the research? | Is a qualitative methodology appropriate? | Was the research design appropriate to address the aims of the research? | Was the recruitment strategy appropriate to the aims of the research? | Was the data collected in a way that addressed the research issue? | Has the relationship between researcher and participants been adequately considered? | Have ethical issues been taken into consideration? | Was the data analysis sufficiently rigorous? | Is there a clear statement of findings? | How valuable is the research? |
| **Dinsdale, 2016** | Yes | Yes | Yes | Yes | Yes | Yes | Yes | Yes | Yes | Valuable |
| **Keely, 2017** | Yes | Yes | Yes | Yes | Yes | No | Yes | Can't tell | Yes | Valuable |
| **Lingetin, 2017** | Yes | Yes | Yes | Can't tell | Yes | Can't tell | Yes | Can't tell | Yes | Can't Tell |
| **Furber, 2011** | Yes | Yes | Yes | Yes | Yes | No | Yes | Yes | Yes | Not valuable |
| **DeJoy, 2016** | Yes | Yes | Yes | Yes | Yes | Yes | Yes | Yes | Yes | Valuable |
| **Keely, 2016** | No | Yes | Can't tell | Can't tell | Can't tell | No | Can't tell | No | Yes | Not valuable |
| **Turato, 2015** | Yes | Yes | Yes | Yes | Yes | No | Yes | Yes | Yes | Valuable |
| **Sui, 2013** | Yes | Yes | Yes | Yes | Yes | Yes | Yes | Yes | Yes | Valuable |
| **Petrov Fieril, 2017** | Yes | Yes | Yes | Yes | Yes | Yes | Yes | Yes | Yes | Valuable |
| **Mills, 2013** | Yes | Yes | Yes | Yes | Yes | No | Yes | Yes | Yes | Can't Tell |
| **Wang, 2015** | Yes | Yes | Yes | Yes | Yes | No | Yes | Yes | No | Can't tell |
| **Keslehurst, 2017** | Yes | Yes | Yes | Yes | Yes | No | Yes | Yes | Yes | Valuable |
| **Jarvie, 2017** | Yes | Yes | Yes | Yes | Yes | No | Yes | Yes | Yes | Valuable |
| **Keely, 2011** | Yes | Yes | Yes | No | Yes | No | Yes | Yes | Yes | Valuable |
| **Furness, 2011** | Yes | Yes | Yes | Yes | Yes | Yes | Yes | Yes | Yes | Not valuable |
| **Nyman, 2010** | Yes | Yes | Yes | Yes | Yes | Yes | Yes | Yes | Yes | Can't tell |
| **Kominiarek, 2015** | Yes | Yes | Yes | No | Yes | No | Can't tell | Yes | Yes | Valuable |
| **Sui, 2013** | Yes | Yes | Yes | Yes | Yes | Yes | Yes | Yes | Yes | Valuable |
| **Weir, 2010** | Yes | Yes | Yes | Yes | Yes | Yes | Yes | Can't tell | Yes | Can't Tell |
| **Denison, 2015** | Yes | Yes | Yes | Yes | Yes | Can't tell | Yes | Yes | Yes | Can't Tell |
| **Faria, 2013** | Yes | Can't tell | Yes | Can't tell | Yes | Can't tell | Can't tell | Can't tell | Yes | Not valuable |
| **Lavendar, 2016** | Yes | Yes | Yes | Yes | Yes | Yes | Yes | Yes | Yes | Valuable |
| **Hastings-Tolsma, 2010** | Yes | Yes | Yes | Yes | Yes | No | Yes | Yes | Yes | Valuable |
| **Lindhart, 2013** | Yes | Yes | Yes | Yes | Yes | Yes | Yes | Yes | Yes | Can't tell |
| **Knight-Agarwal, 2016** | Yes | Yes | Yes | Yes | Yes | Can't tell | Yes | Yes | Yes | Can't Tell |
| **Atkinson, 2017** | Yes | Yes | Yes | Yes | Yes | Yes | Yes | Yes | Yes | Valuable |
| **Khazaezade, 2011** | Yes | Yes | Yes | No | Yes | No | Yes | Can't tell | Yes | Valuable |
| **Holton, 2017** | Yes | Yes | Yes | Yes | Yes | No | Yes | Yes | Yes | Valuable |
| **Heslehurst, 2015** | Yes | Yes | Yes | Yes | Yes | No | Yes | Can't tell | Yes | Valuable |
| **Keenan, 2010** | No | Yes | Yes | Can't tell | Can't tell | No | Yes | Can't tell | Yes | Can't tell |
| **Cunnigham, 2018** | No | No | No | No | No | No | No | No | No | Can't Tell |
