# Supplementary material for: Perceptions of risk and influences of choice in pregnant women with obesity. An evidence synthesis of qualitative research
Source: PLoS One. 2020 Jan 3;15(1):e0227325. doi: 10.1371/journal.pone.0227325 (PMC6941828; doi:10.1371/journal.pone.0227325)
Supplement: S8 Table — (DOCX) [file pone.0227325.s008.docx]

**S8 Table – Full Details of the GRADE-CERQual Assessments**

**Finding 1: Women felt that health-professionals pre-conceived stereotyped beliefs regarding their weight led to over-inflated presentations of risk**

| **Data to support the study finding** | **Methodological limitations** | **Coherence** | **Relevance** |
| --- | --- | --- | --- |
| *Several women, like Jane, felt stigmatised by the oﬀer of a dietary consultation, oﬀended by the suggestion that she needed advice(1).* | Minor concerns - the researchers have not documented consideration of their personal biases | Vague | Women with BMI>40 only |
| *She decided, from my BMI, that they were going to need an electric bed for me. So that they didn’t have to manoeuvre me up and down the bed, and everything. And I didn’t need any help moving myself around. I was up and about and I was off the morphine very quickly. They just presume. Without looking at you, they must have looked at just parts of your notes. Decision taken y I was taken to one side and it was put very nicely, without using the words ‘you’re very fat, and going to be too heavy for our nurses to lift, it would be better all round if we gave you this bed. Because you can manipulate yourself up and down. And my nurses won’t have to struggle because we’re not supposed to’. It’s that assumption because you’re overweight. I thought ‘Yes. That’s somebody stereotyping me’*(2)*.* | Minor concerns - the researchers have not documented consideration of their personal biases | Good fit | BMI 54 |
| *More than one-third of participants reported that providers conflated an elevated risk for certain complications with certainty that the client would develop those complications*(3)*.* | No concerns | No relation to stereotyping | Good fit |
| *more than one-third of participants with otherwise uncomplicated pregnancies described increased medicalization and surveillance of their pregnancy beyond what they felt a thinner woman would receive. Increased scrutiny took the form of perceived excessive or inappropriate dietary counseling, laboratory testing to diagnose gestational diabetes and preeclampsia, and ultrasonography and tests of fetal well-being*(3)*.* |  | Good fit - but this can also be explained by increased risk, not just by stereotyping |  |
| *Women acknowledged if or when problems were present care would need to be modiﬁed, by treating all overweight women it was, as Alison stated, ‘like they pre-empt problems’.* (4) | Minor concerns - the researchers have not documented consideration of their personal biases | Vague | Good fit |
| *Cherry, who perceived herself to be reprimanded by HCPs for not adhering to lifestyle changes/diabetic regimen, reported being informed that she was, ‘going to have a really, really big baby’, and said that additionally she had been told:  …she would be an obese kid, “obese child that’s gonna have diabetes”…you think, “Really love or are you just trying to scare me?”…I think she was in a bad mood that day anyway. I think she was just trying to scare me into managing my diabetes…and because of my age [19] as well*(5) | Minor concerns - the researchers have not documented consideration of their personal biases | Good fit | Women with GDM only |
| *The interviews also indicated that women were aware that excessive weight gain might cause adverse maternal and neonatal health outcomes (10/26 interviews, including ﬁve overweight women and 5 obese women):  'Oh! Not good for your baby. Not good for you…probably difﬁcult labour'. (In03, overweight)* (6) | No concerns | Vague | Good fit |
| *Participants had concerns that they were being judged because of their weight*(7). | No concerns | Explains judgement but not association with over-inflated presentations of risk | White women only |

**Finding 2: Women felt penalised for their weight when health-professionals 'boxed them in' with other obese women.**

| **Data to support the study finding** | **Methodological limitations** | **Coherence** | **Relevance** |
| --- | --- | --- | --- |
| *Alison likened this to being put in a ‘bucket’, ‘you’re overweight “beepbom” you’re in this bucket. It’s sort of like they have a bucket for different people and you don’t feel like you’re in a bucket you just feel like you’re a person’.* (4) | Minor concerns - the researchers have not documented consideration of their personal biases | No evidence that women are penalised by this | Good fit |
| *This corroborates ﬁndings from Furness et al.(2011, p.5), where a participant described feeling penalised because she had to take, ‘the fat girls’ test’*(5). | Minor concerns - the researchers have not documented consideration of their personal biases | Good fit | Women with GDM only |
| *Women felt they were being stigmatised if high BMI was perceived to be the sole reason they were asked to take the Glucose Tolerance Test (for GDM). Some argued the test should be universal in pregnancy:  I don’t think they should immediately look at you and say well ‘because you are like 3 stone overweight, you are more likely to have diabetes than somebody else…They look at you and say ‘you’re overweight’, bang you’re in. They make assumptions….I think in some ways that is a little bit of discrimination you know?…I think the test [GTT] should be standard. [Andrea]* (5). |  | Good fit |  |
| *I felt again like I was being penalised because I was fat. I used to say, ‘oh, I’ve got to do the fat girls’ test again, have I?’ All the time, I felt like they were picking on you because you were fat ... But that’s obviously because I knew I was fat, and I was just ‘I don’t need somebody to tell me that I’m fat, thank you very much!’ (Alice)* (8). | No concerns | Good fit | Good fit |
| *Being ‘labelled’ also made some women defensive and upset, even when they understood ‘with their logical head’ why they were being ‘put into boxes’ because of the related risks*(9)*.* | Minor concerns - the researchers have not documented consideration of their personal biases and the description of the analysis is not in depth | Good fit | Good fit |
| *I never felt labelled as a pregnant lady with a raised BMI, it was always just a "well, this is what the science shows".*(7) | No concerns | Refutes the findings | Good fit |

**Finding 3: An insensitive or stigmatising approach to counselling by health professionals led women to feel ashamed of their weight and blamed by the clinician or themselves for complications which arose.**

| **Data to support the study finding** | **Methodological limitations** | **Coherence** | **Relevance** |
| --- | --- | --- | --- |
| *made to feel ‘naughty’, ‘embarrassed’ or ‘ashamed’ because of their weight, due to healthcare professionals being ‘blunt’ or insensitive*(10)*.* | No concerns | Made to feel ashamed of weight rather than blame for complications | Good fit |
| *Some women recalled concern about being judged in relation to their weight, due to previous personal experiences or friends’ experiences*(10)*.* |  | Not related to current experience |  |
| *within society, or among healthcare professionals there was perceived stigma and assumptions in relation to obesity, and expressed concern about being judged because of their weight*(10). |  | Judgement of weight rather than blame for complications |  |
| *This evoked feelings of guilt and self-blame that she should have lost weight before conceiving as she was concerned that her obesity may harm the infant: She’d told me that she was ﬁnding it hard to ﬁnd the baby’s heartbeat because I was overweight. I come out and I was in ﬂoods of tears. You think that you’re doing the baby some wrong. Where you weren’t in a ﬁt state when you started it, and you should have been*(2). | Minor concerns - the researchers have not documented consideration of their personal biases | Good fit | BMI>35 only |
| *self-blame and guilt for placing their fetus at risk due to their weight*(3). | No concerns | Good fit for blame but not direct link to an insensitive approach from the healthcare professional. | Good fit |
| *Some women perceived themselves to be blamed by HCPs for having a deﬁcient lifestyle (see also Nicklas et al., 2011), ‘I felt like straightaway they were saying, ‘Because of the foods you are eating you have caused yourself to get gestational diabetes’ [Fiona]. Participants discussed having to show HCPs their dietary records, with some saying they felt judged, and/or disbelieved*(5). | Minor concerns - the researchers have not documented consideration of their personal biases | Good fit | Women with GDM only |
| *All women were aware of their risk of having a big baby. Many participants were anxious that having a big baby was potentially stigmatising, and a site of maternal blame (see also, Jarvie, 2016). It worried me that I’d have a big baby and that it was my fault…I suppose it’s a bit like stigma isn’t it? If you’ve got a big baby it’s not seen as a good thing is it?’ [Fiona]*(5). |  | Good fit |  |
| *A number of women reported being informed by HCPs that GDM was directly attributable to their weight. Some accepted this, others felt unjustly blamed: It’s like, ‘this [GDM] is pretty much your fault because you are overweight’. It all comes down to weight, not, ‘Oh it could be just that your body doesn’t sort out sugar enough’. I mean anybody could have it. [Gemma]* (5). |  | Good fit |  |
| *I think there is an element that probably for most people, their status as a pregnant women with a raised BMI is, to a certain extent, sort of self-inflicted and, you know, the needing of additional care as simply as a result of that does, year, definitely made me feel guilty’*(7). | No concerns | Good fit | Good fit |

**Finding 4: The way obese women perceived risk in pregnancy was heavily influenced by the nature or antenatal counselling received (or lack thereof).**

| **Data to support the study finding** | **Methodological limitations** | **Coherence** | **Relevance** |
| --- | --- | --- | --- |
| *women perceiving that weight was discussed too much*(10)*.* | No concerns | Doesn't support a link with perception of risk | Good fit |
| *Women perceived being told about risks positively, preferring healthcare professionals to be honest and upfront*(10)*.* |  | Good fit | Good fit |
| *wanting constructive advice about how to minimise these risks*(10)*.* |  | Good fit |  |
| *they were giving me ways to solve it, which was good*(10). |  | Good fit | BMI>40 |
| *Some women appreciated the reinforcement of their knowledge by a health professional (expert) as it increased their conﬁdence, whereas others considered this to be wasted time that could have been spent discussing new pregnancy speciﬁc information, such as linking diets to fetal development*(11)*.* | Minor concerns - the researchers have not documented consideration of their personal biases | Good fit | Good fit |
| *Ultrasound scans were experienced as distressing when women were informed their high BMI might compromise visualisation of the fetus (see also Furber and McGowan, 2010). Gemma commented:  I mean it did upset me when they were like, ‘Oh yeah it’s because you’re overweight’ and ‘You’re rather large’ and ‘We actually can’t see properly because you are obese’…I didn’t want [partner] to come in because of that. I knew they were going to bring it up because it gets brought up every single week*(5)*.* | Minor concerns - the researchers have not documented consideration of their personal biases | Good fit | Women with GDM only |
| *“The GP weighed me and measured my BMI without even speaking to me about it. Then I am referred to a special practice for fat pregnant women without my consent. I really feel stigmatized. It is quite all right to speak about smoking but why can they not address the obesity as it is. I am much more upset now than if he had told me up front. I’m going to confront him next time I see him”* (12)*.* | No concerns | Good fit | Good fit |
| *“I was so excited, after years of struggling to become pregnant, I ﬁnally met my midwife. After approximately 10 minutes into the conversation, she suddenly said. ‘Don’t expect to be able to breastfeed when your BMI is so high’. I felt slammed up against the wall, did not really hear more of the conversation. I was devastated”* (12)*.* |  | Good fit | Good fit |
| *This was illustrated in an experience of Olive’s, who following an antenatal consultation a healthcare professional said ‘straight to my face, you don’t want to have a dead baby do you’? (Olive: BMI 48). This experience created a great deal of anxiety for Olive throughout her pregnancy particularly as she had a previous miscarriage. ‘So through my whole pregnancy it was hard like do you know what I mean….I was thinking all that and two years ago I had a miscarriage so I had that in the back of my mind and then the nurse said to me you don’t want to have a dead baby like so, what went through my head, through the rest of my pregnancy…. the baby was going to die’ (Olive: BMI 48)* (13)*.* | No concerns | Good fit | BMI 48 |
| *I think that the midwives and doctors at [the hospital] don’t make a huge deal about weight … they don’t emphasise your weight but they do tell you the risks. (Ruby, BMI ≥ 30 kg/m2)* (14)*.^.^* | Minor concerns - the researchers have not documented consideration of their personal biases | Doesn't specify how the healthcare professional approached the risk counselling. | Good fit |

**Finding 5: Women who normalise potential risks in pregnancy do this in response to a belief that risks are either unrelated to their obesity, or to their perception that they themselves are healthy.**

| **Data to support the study finding** | **Methodological limitations** | **Coherence** | **Relevance** |
| --- | --- | --- | --- |
| *Almost all of the pregnant participants described themselves as ‘healthy’, despite their weight. Some attempted to normalise their weight in order to relieve anxiety regarding pregnancy risk*(1)*.* | Minor concerns - the researchers have not documented consideration of their personal biases | Good fit | Women with BMI>40 only |
| *highlighting the positive pregnancy experiences of family, friends or those accessed online who also had a raised BMI in pregnancy*(1)*.* |  | Vague |  |
| *I’d only just made it into their clinic. That I was one of the smallest ladies that she treats…The last time I saw her in the clinic, she made it pretty clear that she didn’t expect to ever see me again. She gave me my plan of care: ‘Go and have a nice delivery’*(1)*.* |  | Vague |  |
| *Other participants, both pregnant women and partners, drew on ecological fallacies in order to negotiate the notion of weight-related pregnancy risk. These included: highlighting other health behaviours, such as drinking or smoking in pregnancy, as more risky than having a high BMI*(1). |  | Down-plays the role of obesity in increasing risks |  |
| *emphasising pregnancy as by its nature risky, and the experience of complications as randomly occurring among the pregnant population*(1). |  | Disassociates obesity with the increase risk profile |  |
| *A number of women reported being informed by HCPs that GDM was directly attributable to their weight. Some accepted this, others felt unjustly blamed: It’s like, ‘this [GDM] is pretty much your fault because you are overweight’. It all comes down to weight, not, ‘Oh it could be just that your body doesn’t sort out sugar enough’. I mean anybody could have it. [Gemma]* (5). | Minor concerns - the researchers have not documented consideration of their personal biases | Good fit | Women with GDM only |
| *The midwives that told me about the diabetes said that. I think they believe anything they read as well. Like they are saying like all these scientiﬁc experiments show this and all that lot, but I don’t think some of it’s true…*(5). |  | Denial of risk / misinformation |  |
| *Seven of the eight women interviewed perceived themselves as generally healthy. In addition, many felt their weight had not had a negative effect on their health in pregnancy:  ‘I’m still as healthy as anybody else that’s pregnant. I’m just carrying a bit more weight. That’s how I perceive it as’ (Mary, Gravida 3 Para 1 (G3P1), previous 2nd trimester loss 17/40, BMI 44)* (15)*.* | Minor concerns - the researchers have not documented consideration of their personal biases | Good fit | Women with BMI>40 only |
| *Some of the women who described their health as good also described complex medical and obstetric histories; in some cases these were due to conditions and complications which may have been caused by obesity*(15)*.* |  | Good fit |  |
| *When the patients viewed a chart of BMI’s and normal ranges of weight based on height, they expressed strong opinions against the chart (e.g., “It’s a lie”) and that their weight did not increase health risks since women with normal weights could still have problems such as diabetes or hypertension and hereditary factors or a family history could account for health risks*(16)*.* | Moderate concerns - the researchers have not documented consideration of their personal biases and the aim was to sample majority minority women but the sampling strategy did not reflect this. | Good fit | Non-Hispanic black women from USA only |

**Finding 6: Most women and healthcare professionals avoid counselling regarding risks in pregnancy associated with obesity.**

| **Data to support the study finding** | **Methodological limitations** | **Coherence** | **Relevance** |
| --- | --- | --- | --- |
| *In the absence of an explanation women described finding out about risks from friend’s experiences or via the internet*(10)*.* | No concerns |  | Good fit |
| *There were some women on pathways 1 or 2 who felt their weight had been ‘skimmed over’, or written in notes but never discussed*(10). |  |  |  |
| *“On one of my scans it said ‘difficult viewing because of obesity’…but no one ever mentioned it. I read it and I was like, oh God! But I was pleased that no one actually said it. But I suppose they shouldn’t skirt around it… (they should) be more supportive, and bring it up. I think they are too polite almost; they daren’t say it”* (10)*.* |  |  | Good fit |
| *not aware of specific risks due to their BMI, due to a lack of explanation..* (10). |  |  | Good fit |
| *Vicky described issues with the referral process, and how her midwife gave her the leaﬂet about obesity-related risks for the mother and baby at booking as part of the referral process, with no explanation of the content of the leaﬂet or in person risk communication*(11)*.* | Minor concerns - the researchers have not documented consideration of their personal biases |  | Good fit |
| *Fiona had asked her midwife to record in her notes a plea for HCPs to desist from making further weight-related comments because it was, ‘ruining her pregnancy’*(5)*.* | Example of women also wishing to avoid discussion |  | Women with GDM only |
| *‘I’ve probably just kinda ... maybe read them and then blocked them out again (laughs). [I] try not to think about them too much. I know if I think about them too much I’ll start to worry ... and that will eventually make it all worse’ (Diane, G1P0, previous miscarriage 6/40, BMI 42)* (15)*.* | Example of women also wishing to avoid discussion |  | Women with BMI>40 only |
| *‘It was kind of weird actually. It hadn’t been raised at all until I got a phone call from the midwife saying, ‘I’ve got two scans and an appointment with a consultant for you’. I was like, ‘What’s wrong, like?’ And then she said ‘No, no, it’s just because you’re obviously … a BMI over 40’ (Fiona, G1P0, BMI 42)* (15)*.* | Minor concerns - the researchers have not documented consideration of their personal biases |  |  |
| *‘It probably is something that’s kind of been skirted around a bit, only for reasons of maybe ... errm, other issues, I mean I was advised to wear the TEDS [surgical] stockings and things like that. So, I mean there are connections back to the fact that I’m overweight, but nothing in specific terms’ (Kim, G2P1, BMI 46)* (15)*.* |  |  |  |
| *Women knew that they were obese and expected to discuss this when they attended their antenatal booking visit. However, information received from health professionals lacked information regarding their weight and gestational weight management*(17)*.* | No concerns |  | Women attending a postnatal lifestyle programme |
| *when I booked in .. . with my midwife, she never really mentioned anything about my weight .. . and all I’d read up was what was in baby magazines that you know, some overweight mothers do have complications.... (BMI of 38.9 kg/m2, aged 21)* (17). |  |  | BMI 38.9 |
| *With only one exception, all participants were unaware that they had been referred by their GP to the specialist midwifery clinic for pregnant women with obesity*(12)*.* | No concerns |  | Good fit |
| *Most of the participants were informed by their GP that they had to have an oral glucose tolerance test and that being obese during pregnancy may cause disturbances in the blood sugar level. When they asking further about what might happen to themselves or their child, none of the women was informed of other consequences of having obesity during pregnancy*(12)*.* |  |  |  |
| *Other women recognised that talking about weight is a sensitive issue and that this may be a reason why some health professionals ‘shy away’ from such conversations. One participant commented: ‘‘I mean I suppose maybe there is. . . . . . a little bit of shying away from that um with pregnancies now, maybe because I think too much of an emphasis can make you feel awful, and terrible the whole time’’*(18)*.* | Minor concerns - the researchers have not documented consideration of their personal biases |  | Good fit |
| *When asked if information or advice was given regarding their BMI all participants recalled having their weight taken to calculate their BMI. However, they also indicated a lack of information received regarding increased BMI or weight management from the health professionals. ‘It was never mentioned at all either this time or my ﬁrst pregnancy*(13)*.* | No concerns |  | Good fit |
| *One participant appeared somewhat bemused that weight was not  discussed and described that her BMI was ‘just recorded and that’s all’ with no explanation given by any healthcare professional. ‘I was weighed yes, and that’s all. And that’s all… not to, nobody say it’s too big or too small or… just recorded and that’s all’ (laughter) (Breda: BMI 31)* (13)*.* |  |  | Good fit |
| *A number of participants speciﬁed other issues that were given greater attention such as blood pressure, domestic abuse or smoking. ‘I think, to be honest the blood pressure was probably the main thing that was checked and that was looked at and if that was normal or good or ﬁne … (Beth: BMI 32). ‘It was more about abuse questions, is what they were more interested in than being overweight to be honest’ (Gina: BMI 35)* (13)*.* |  |  | Good fit |
| *I think that she just found it a little bit awkward and didn't really like talking about it, but then I didn't ask questions about it either’*(7)*.* | No concerns | Good fit | Good fit |
| *There has not been any discussion. There has been no discussion at all about my weight, um, whether being overweight, or you know, the amount of weight I have put on in pregnancy there hasn't been any, no discussion about it all'*(7)*.* |  |  |  |

**Finding 7: A lack of counselling regarding potential complications of pregnancy associated with obesity causes women to feel unprepared or shocked when presented with risk.**

| **Data to support the study finding** | **Methodological limitations** | **Coherence** | **Relevance** |
| --- | --- | --- | --- |
| *discussion of risks only occurring when a complication arose*(10)*.* | No concerns | Vague | Good fit |
| *For Sophie and her daughter Diane, hearing Sophie described as ‘high risk’, by the receptionist in the antenatal clinic, made them anxious and frightened. I went there (reception) and the lady said to me ‘oh, she’s on high risk’ and I was shocked... because they (doctor) didn’t mention anything...he said he’s concerned about my age...* (3). | Minor concerns - the researchers have not documented consideration of their personal biases | Good fit | BMI 37 |
| *‘I watched this stupid programme on telly that I shouldn’t have done ... the one with the big babies and obese mothers. That was a big shock. It was really, really obese people and how it gives them a higher chance of having big babies and the health problems that that ... you know, it was babies over a stone and stuff ... it was pretty grim. And I’m getting bracketed in with all these people because of my weight ... so that was a big shock’ (Emma, G2P1, BMI 40)* (19)*.* | Minor concerns - the researchers have not documented consideration of their personal biases | Good fit | BMI 40 |
| *“The GP weighed me and measured my BMI without even speaking to me about it. Then I am referred to a special practice for fat pregnant women without my consent. I really feel stigmatized. It is quite all right to speak about smoking but why can they not address the obesity as it is. I am much more upset now than if he had told me up front. I’m going to confront him next time I see him”* (12)*.* | No concerns | Doesn't specify feeling unprepared/shocked. Reflects more on a feeling of stigmatisation. | Good fit |
| *encounters with healthcare professionals became an unpleasant experience and caused distress, as she had not been informed beforehand that it might be difﬁcult to scan an obese individual*(12)*.* |  | Good fit | Good fit |

**Finding 8: A lack of discussion regarding the potential complications of pregnancy provides false reassurance to some obese women**

| **Data to support the study finding** | **Methodological limitations** | **Coherence** | **Relevance** |
| --- | --- | --- | --- |
| *This lack of discussion led some women to believe that they were having a ‘normal pregnancy’ (Woman with a BMI of 38.9 kg/m2, aged 21); believing that they were at low risk of complications lulled them into a false sense of security: I’ve never been told before either by my doctor, or anything, that I needed to lose weight either which has surprised me because I’m like really overweight,...so they must have thought, right, well, you’re fairly healthy, you’re just overweight (BMI of 50.1 kg/m2, aged 24)* (17)*.* | No concerns | Good fit | Women attending a postnatal lifestyle programme |
| *Yeah, it felt like she didn’t think I was a great risk and, you know, I wasn’t that much of a problem..... if it was a problem to me, she didn’t think it was a problem, thank God (BMI of 38.9 kg/m2, aged 21)* (17)*.* |  | Good fit |  |
| *As a result of the health professionals not speaking about pregnancy BMI or gestational weight gain this apparently implied to the women that weight was not a priority*(18)*.* | Minor concerns - the researchers have not documented consideration of their personal biases | Good fit | Good fit |
| *However, the doctor implied that the BMI recorded on the notes was in fact lower than the actual calculated BMI.’She (doctor) was calculating my BMI, I think she said it was 30 or 32 and she goes, now that’s not so bad, thinking about all the weight I had put up, now she said ‘I’m being generous with you – I am being nice to you’, whatever way she was calculating it (BMI) was probably more (Dora: BMI 31)* (13)*.* | No concerns | Vague | Good fit |

**Finding 9: Women who accept the potential for pregnancy risks proceed through pregnancy with anxiety or fear for the occurrence of complications.**

| **Data to support the study finding** | **Methodological limitations** | **Coherence** | **Relevance** |
| --- | --- | --- | --- |
| *fear of complications*(3)*.* | No concerns | Good fit | Good fit |
| *The lack of explanation about the service at the point of referral made women feel fearful and nervous about attending the initial session*(11)*.* | Minor concerns - the researchers have not documented consideration of their personal biases | Related to lack of explanation rather than potential risks | Good fit |
| *All women were aware of their risk of having a big baby. Many participants were anxious that having a big baby was potentially stigmatising, and a site of maternal blame*(5). | Minor concerns - the researchers have not documented consideration of their personal biases | Good fit | Women with GDM only |
| *Fiona had asked her midwife to record in her notes a plea for HCPs to desist from making further weight-related comments because it was, ‘ruining her pregnancy’*(5)*.* |  | Vague |  |
| *Ultrasound scans were experienced as distressing when women were informed their high BMI might compromise visualisation of the fetus (see also Furber and McGowan, 2010). Gemma commented:  I mean it did upset me when they were like, ‘Oh yeah it’s because you’re overweight’ and ‘You’re rather large’ and ‘We actually can’t see properly because you are obese’…I didn’t want [partner] to come in because of that. I knew they were going to bring it up because it gets brought up every single week*(5)*.* |  | Vague |  |
| *‘I’ve probably just kinda ... maybe read them and then blocked them out again (laughs). [I] try not to think about them too much. I know if I think about them too much I’ll start to worry ... and that will eventually make it all worse’ (Diane, G1P0, previous miscarriage 6/40, BMI 42)* (19)*.* | Minor concerns - the researchers have not documented consideration of their personal biases | Good fit | Women with BMI>40 only |
| *In the light of this awareness of the risks, many of the women expressed anxiety:  ‘You know, epidurals and stuff like, because I know there’s obviously higher risks, you know, with things like that’*(19)*.* |  | Good fit |  |
| *there was one thing I was reading about… shoulder dystocia…which kind of scared the heck out of me’*(19)*.* |  | Good fit |  |
| *And just a whole lot of other health conditions that comes along that really, really triggers some stress*(16)*.* | Moderate concerns - the researchers have not documented consideration of their personal biases and the aim was to sample majority minority women but the sampling strategy did not reflect this. | Good fit | Non-Hispanic black women from USA only |
| *This was illustrated in an experience of Olive’s, who following an antenatal consultation a healthcare professional said ‘straight to my face, you don’t want to have a dead baby do you’? (Olive: BMI 48). This experience created a great deal of anxiety for Olive throughout her pregnancy particularly as she had a previous miscarriage. ‘So through my whole pregnancy it was hard like do you know what I mean….I was thinking all that and two years ago I had a miscarriage so I had that in the back of my mind and then the nurse said to me you don’t want to have a dead baby like so, what went through my head, through the rest of my pregnancy…. the baby was going to die’ (Olive: BMI 48)* (13)*.* | No concerns | Good fit | BMI 48 |
| *Women felt they understood the potential risks to their baby due to their current weight which caused them to worry*(9)*.* | Minor concerns - the researchers have not documented consideration of their personal biases and the description of the analysis is not in depth | Good fit | Good fit |

**Finding 10: Some women, who accept the potential for risk in pregnancy, consider such risks to be inevitable and their occurrence to be out of their control.**

| **Data to support the study finding** | **Methodological limitations** | **Coherence** | **Relevance** |
| --- | --- | --- | --- |
| *Emphasising pregnancy as by its nature risky, and the experience of complications as randomly occurring among the pregnant population*(1)*.* | Minor concerns - the researchers have not documented consideration of their personal biases | Good fit | Women with BMI>40 only |
| *Although the women generally had an increased awareness of  the importance of weight management in pregnancy, there was a collective lack of speciﬁc awareness of the risks of excessive weight for the mother and baby, and an acceptance that weight gain is inevitable, natural and acceptable during pregnancy: ‘you can only control your weight up to a certain point in pregnancy [laughing] because it’s gonna happen anyway.’ (NP, BMI 42 kg/m2)* (20)*.* | Minor concerns - the researchers have not documented consideration of their personal biases | Good fit | Women with BMI>40 only |

**Finding 11: Some obese women often felt forgotten about during their antenatal care, with the needs of their unborn baby often prioritised above their own needs.**

| **Data to support the study finding** | **Methodological limitations** | **Coherence** | **Relevance** |
| --- | --- | --- | --- |
| *Several women noted that the focus was on fetal well-being and screening of the women, rather than the mother and the infant as a whole*(2)*.* | Minor concerns - the researchers have not documented consideration of their personal biases | Good fit | Women with BMI>35 |
| *In many ways, don’t get me wrong I wouldn’t do anything to harm the baby at all, but it’s like the baby comes first and you are like an oven.And that’s how I feel. That as long as I can manage, even if I’m suffering, it’s fine, because the baby’s going to be fine.It all seems to be geared to that. So what they’ve done is denied me the right to my health, for the sake of the baby*(2)*.* |  | Good fit | BMI 54, GDM |
| *Ultrasounds were problematic with the main area of concern ‘with them being able to feel where the baby is’ (Anita); this was particularly notable earlier in the pregnancy when the baby was smaller. Women commented about the amount of pressure applied through the transducer and the pain and discomfort this caused, ‘she was digging into my ribs, and I went, yeah I know there’s a baby there too, but still – it’s still me’ (Leah).Leah expressed concern about feeling she had been forgotten in the process of focusing on the procedure*(4)*.* | Minor concerns - the researchers have not documented consideration of their personal biases | Good fit | Good fit |
| *‘He (doctor) would have said, you know, ‘sleep for two, eat for one’, is his moto if you are lik’…but just watch what I was eating from the point of view of having the baby being too big, but not from a BMI point of view, I suppose it was more from the baby’s size point of view actually’ (Beth: BMI 32)* (13). | No concerns | Good fit | Good fit |

**Finding 12: Stresses with women's family and professional lives influence choices that they made regarding their antenatal care.**

| **Data to support the study finding** | **Methodological limitations** | **Coherence** | **Relevance** |
| --- | --- | --- | --- |
| *Convenience was another key factor, the midwives clinics had ﬂexible hours ‘my partner can’t make these appointments...you only get certain days you can come because you’re overweight’ (Alison); similarly Therese was unable to get care for her other children during the day, and in school holidays had to bring seven children to the antenatal clinic while she attended the doctors clinic, which often involved waiting for long periods in a busy environment*(4)*.* | Minor concerns - the researchers have not documented consideration of their personal biases | Good fit | Good fit |
| *Some women questioned the necessity for such frequent attendance at hospital diabetic antenatal clinics, and expressed a desire for more of their care to be carried out in the community/by their community midwife/over the telephone.  I don’t think I needed to go to hospital as much as I did…A trip to [hospital 20 miles away], it’s not cheap. And I was having to take time oﬀ work as well. I don’t think I needed to go as much as it was. To be honest with you I think you should see your midwife and check the readings [blood glucose levels (BGLs)] and if it’s higher then sending you to hospital is all that’s really needed. [Fiona]* (5). | Minor concerns - the researchers have not documented consideration of their personal biases | Good fit | Women with GDM only |
| *Many participants emphasised the expenditure required to attend  additional antenatal appointments due to obesity/diabetes. Women/ families on beneﬁts/low incomes struggled to meet transportation and hospital parking costs. Working women worried about employers not being favourable to time oﬀ. Some women paid for additional childcare to avoid being accompanied by young children to lengthy clinic appointments*(5)*.* |  | Good fit |  |
| *Many women in the study were experiencing acute and/or chronic  stress during pregnancy and the post-birth period. Stressful events such as redundancy, bereavement, relationship breakdown, eviction and cessation of beneﬁt payments induced acute stress. Day-to-day stressors included ﬁnancial worries, housing insecurities, overcrowded/poor quality housing, caring for sick/elderly relatives, isolation and coping with young children with little/no support. Social and economic stressors/constraints aﬀected women’s ability to: cope during pregnancy/post-birth; make lifestyle changes; attend antenatal appointments; manage their weight*(5)*.* |  | Good fit |  |

**Finding 13: Relationships with healthcare providers which were perceived negatively by the women made them feel as if they had no choice with regards to their pregnancy and birth**

| **Data to support the study finding** | **Methodological limitations** | **Coherence** | **Relevance** |
| --- | --- | --- | --- |
| *Not liking injections, not the best sort of meeting for me. I really did feel she came across as telling us the negative sides of it. What could go wrong, so we came away thinking we’re not having an epidural, and that’s my decision now. I’m not having one*(2)*.* | Minor concerns - the researchers have not documented consideration of their personal biases | Vague - decision might have been made due to concerns re. potential risks | BMI 38 |
| *others felt uncomfortable challenging their provider due to fear of what might happen to them or their fetuses if they did not follow medical advice.^27^* | No concerns | Good fit | Good fit |
| *A couple of participants reported that they felt coerced into giving birth via cesarean: “I was not preeclamptic; I wasn’t diabetic; I wasn’t having any additional problems; I had no complications. I was just fat while pregnant and therefore needed a c-section”* (3)*.* |  | Good fit |  |
| *I didn’t want to go see this doctor at all. After the second or third appointment where she kept on harping on, “You can’t deliver this baby, you’re too fat to deliver this baby, you need a repeat c-section,” I just didn’t want to go. It was upsetting enough that, you know, I didn’t attempt to sabotage the appointments, but I would leave the house late, or I would drag my feet getting ready to leave the house because I didn’t want to be subjected to another appointment*(3)*.* |  | Vague |  |
| *She was more worried about the humiliating treatment she  might receive than about how the baby was doing; something that was noted in her ﬁle. At a later visit Irma was told about this note, but otherwise ignored. She was told ‘notwithstanding this, the fact is that you really are obese’. As she was afraid of not getting adequate care, she did not dare to question this humiliating treatment*(3)*.* |  | Good fit |  |
| *I haven’t actually gotten to the point of skipping an appointment. . . . My husband and I considered having this pregnancy be unassisted, and even delivering unassisted. The reason I was considering that was just to avoid appointments. . . . In previous pregnancies, I any problems. Oftentimes I’ve skipped the 6-week checkup. I haven’t had well-woman care because of it [insensitive treatment]* (3)*.* |  | Vague - negative relationship might push women towards a choice of not engaging with antenatal care |  |
| *Gemma also discussed non-attendance of some hospital clinic appointments due to anticipation of further stigmatising experiences*(5)*.* | Minor concerns - the researchers have not documented consideration of their personal biases | Vague - negative relationship might push women towards a choice of not engaging with antenatal care | Women with GDM only |
| *When caregivers left the room and were out of earshot, the  women felt suspicious about what the caregivers thought and said about them, for example how big and difﬁcult to examine they were. Feelings of resignation and a sense of not wanting to jeopardise the situation and risk receiving inadequate care by confronting the caregivers to state their opinions were experienced*(21)*.* | Moderate concerns - very little data to support the findings | Good fit | Good fit |
| *Anxiety about hurting a midwife’s feelings prevented the women from changing to another midwife, even if the woman felt insecure, not recognised or was treated badly*(21)*.* |  | Good fit |  |
| *The participants appeared happy to follow the recommendations of the midwives, despite not having a full understanding of the reasons: 'Were reasons given? No, no particularly. I just did it [the oral glucose tolerance test]; to be honest, I didn't really question it’*(7)*.* | No concerns | Vague | Good fit |

**Finding 14: Women perceived guidelines to be restrictive of their choices**

| **Data to support the study finding** | **Methodological limitations** | **Coherence** | **Relevance** |
| --- | --- | --- | --- |
| *Approximately one-quarter of the participants described difficulty accessing the provider or type of birth that they desired. A couple of participants who wanted midwifery care were transferred to obstetric care, even with no medical complications. Another 2 participants wanted to attempt a vaginal birth after cesarean and were discouraged or refused because of their size*(3)*.* | No concerns | Vague | Good fit |
| *Therese, who was having her eighth child, was frustrated she had to attend the doctor’s clinic rather than the midwives clinic:  they can see that through all my pregnancies so far there’s been no complications...I guess it’s just the criteria that they have down there...you had to be under 100 kilos, and they accepted me at 99, where they wouldn’t accept me this time ‘cos I was 102*(4)*.* | Minor concerns - the researchers have not documented consideration of their personal biases | Good fit | Good fit |
| *Women identiﬁed problems around access to clinical services and equipment that arose as a direct result of their being overweight. ‘It’s just the criteria’ treats me differently. Women in this study were aware decisions about who could attend midwifery-led clinics and the birth centre were guided by hospital policy. Their comments, however, reﬂected concerns about how reliance on guidelines for such decisions led to a loss of individuality and lack of capacity by professionals to assess health concerns or risks on an individual basis*(4)*.* |  | Good fit | Good fit |
| *At the same time Alison stressed she did not feel staff chose to treat her differently to other women; she felt hospital protocols or policies determined the differences in care, she remarked ‘the people I see don’t [choose to treat me differently], it’s just the protocol that they’ve got to’*(4)*.* |  | Good fit | BMI 58 |
| *Helplessness and disappointment were experienced in encounters with caregivers because they did not focus on the women’s needs. A sense of not being understood arose when caregivers required postures that were almost impossible to achieve due to the obese pregnant body*(21)*.* | Moderate concerns - very little data to support the findings | Doesn't directly support the conclusion | Good fit |
| *Not being involved in their care or being disrespected about birth plans evoked feelings of being ignored*(21)*.* |  |  |  |

**Finding 15: Women who perceived their relationship with a healthcare provider positively felt empowered to make choices regarding their pregnancy and birth.**

| **Data to support the study finding** | **Methodological limitations** | **Coherence** | **Relevance** |
| --- | --- | --- | --- |
| *It was amazing to work with medical professionals who didn’t judge me because of my size and really empowered me to believe in my body because they believed in it. That was the first time I had ever had a medical professional uplift me in that way. It [childbirth] was the most amazing experience of my life. After that, I had a whole new appreciation for my body*(3)*.* | No concerns | Good fit | Good fit |
| *The experience of participation in their own care decreased discomfort*(21)*.* | Moderate concerns - very little data to support the findings | Vague | Good fit |

**Finding 16: Women's perceptions of risks influenced the choices that they made regarding their labour and birth.**

| **Data to support the study finding** | **Methodological limitations** | **Coherence** | **Relevance** |
| --- | --- | --- | --- |
| *Not liking injections, not the best sort of meeting for me. I really did feel she came across as telling us the negative sides of it. What could go wrong, so we came away thinking we’re not having an epidural, and that’s my decision now. I’m not having one*(2)*.* | Minor concerns - the researchers have not documented consideration of their personal biases | Good fit | BMI 38 |
| *the knowledge that their infant may be large caused concern. One woman illustrated this with her attempts to stimulate labour early, rather than await the natural onset of labour, when she was aware that the infant could be even heavier at birth a few weeks after the estimated weight at 36 weeks of gestation*(2)*.* |  | Good fit | Women with BMI>35 |
| *The postnatal data also revealed that fetal weight estimates from ultrasound assessments had affected decision-making over mode of birth in the pregnancy period. Participant G, for example, requested an elective caesarean section as she expected her infant to be considerably bigger than he was at birth*(2)*.* |  | Good fit |  |
| *Those with prior pregnancies described how previous experiences with maternity care influenced their expectations for treatment with this pregnancy. Some women in the study also reported that their current experiences would set the tone for future pregnancies*(3)*.* | No concerns | Vague | Good fit |
| *Women wanted the option of seeing the midwives for a number of reasons ‘because I’m after a more natural sort of birth’*(4)*.* | Minor concerns - the researchers have not documented consideration of their personal biases | Good fit - positive example | Good fit |
| *In Fiona’s case this lack of discussion of the risks of obesity early in her pregnancy led to her feeling that the information she was given by her midwives and the anaesthetist was contradictory. She described feeling confused and frustrated following her consultation with the consultant anaesthetist, who had advised her to have an epidural early in her labour*(15)*.* | Minor concerns - the researchers have not documented consideration of their personal biases | Vague - no clear link to choices made | BMI 42 |
| *Most pregnant women relied on family and friends as a powerful primary source of information about sterilization that they perceived as accurate and reliable, even though some of the information (as reported in interviews) was factually incorrect. This was particularly notable regarding the notion that the procedure was “not really permanent,” and that it could be “undone” if the patient changed her mind and desired additional children: My girlfriend had her tubes tied and then had them untied because she decided she wanted another kid*(22)*.* | Minor concerns - the researchers have not documented consideration of their personal biases | Good fit | Women who choose to opt for sterilisation during pregnancy |
| *Nevertheless, these women recognised that being overweight can contribute to maternal pregnancy complications such as an  increased risk of caesarean section as one woman commented: ‘‘I really want to go natural but because of my weight I might not be able to go natural. . . it might be too much of a health risk”* (18)*.* | Minor concerns - the researchers have not documented consideration of their personal biases | Good fit | Good fit |

**References**

1. Keely A, Cunningham-Burley S, Elliott L, Sandall J, Whittaker A. "If she wants to eat...and eat and eat...fine! It's gonna feed the baby": Pregnant women and partners' perceptions and experiences of pregnancy with a BMI >40kg/m(2). Midwifery. 2017;49:87-94.

2. Furber CM, McGowan L. A qualitative study of the experiences of women who are obese and pregnant in the UK. Midwifery. 2011;27(4):437-44.

3. DeJoy SB, Bittner K, Mandel D. A Qualitative Study of the Maternity Care Experiences of Women with Obesity: "More than Just a Number on the Scale". J Midwifery Womens Health. 2016;61(2):217-23.

4. Mills A, Schmied VA, Dahlen HG. 'Get alongside us', women's experiences of being overweight and pregnant in Sydney, Australia. Matern Child Nutr. 2013;9(3):309-21.

5. Jarvie R. Lived experiences of women with co-existing BMI >= 30 and Gestational Diabetes Mellitus. Midwifery. 2017;49:79-86.

6. Sui Z, Turnbull DA, Dodd JM. Overweight and obese women's perceptions about making healthy change during pregnancy: a mixed method study. Matern Child Health J. 2013;17(10):1879-87.

7. Cunningham J, Endacott R, Gibbons D. Communication with health professionals: The views of pregnant women with a raised BMI. British Journal of Midwifery. 2018;26(9):598-604.

8. Furness PJ, McSeveny K, Arden MA, Garland C, Dearden AM, Soltani H. Maternal obesity support services: a qualitative study of the perspectives of women and midwives. BMC Pregnancy Childbirth. 2011;11:69.

9. Heslehurst N, Russell S, Brandon H, Johnston C, Summerbell C, Rankin J. Women's perspectives are required to inform the development of maternal obesity services: a qualitative study of obese pregnant women's experiences. Health Expect. 2015;18(5):969-81.

10. Dinsdale S, Branch K, Cook L, Shucksmith J. "As soon as you've had the baby that's it..." a qualitative study of 24 postnatal women on their experience of maternal obesity care pathways. BMC Public Health. 2016;16:625.

11. Heslehurst N, Dinsdale S, Brandon H, Johnston C, Summerbell C, Rankin J. Lived experiences of routine antenatal dietetic services among women with obesity: A qualitative phenomenological study. Midwifery. 2017;49:47-53.

12. Lindhardt CL, Rubak S, Mogensen O, Lamont RF, Joergensen JS. The experience of pregnant women with a body mass index >30 kg/m(2) of their encounters with healthcare professionals. Acta Obstet Gynecol Scand. 2013;92(9):1101-7.

13. Atkinson S, McNamara PM. Unconscious collusion: An interpretative phenomenological analysis of the maternity care experiences of women with obesity (BMI>/=30kg/m(2)). Midwifery. 2017;49:54-64.

14. Holton S, East C, Fisher J. Weight management during pregnancy: a qualitative study of women's and care providers' experiences and perspectives. BMC Pregnancy Childbirth. 2017;17(1):351.

15. Keely A, Gunning M, Denison F. Maternal obesity in pregnancy: Women’s understanding of risks. British Journal of Midwifery. 2011;19(6):364-9.

16. Kominiarek MA, Gay F, Peacock N. Obesity in Pregnancy: A Qualitative Approach to Inform an Intervention for Patients and Providers. Matern Child Health J. 2015;19(8):1698-712.

17. Lavender T, Smith DM. Seeing it through their eyes: a qualitative study of the pregnancy experiences of women with a body mass index of 30 or more. Health Expect. 2016;19(2):222-33.

18. Knight-Agarwal CR, Williams LT, Davis D, Davey R, Shepherd R, Downing A, et al. The perspectives of obese women receiving antenatal care: A qualitative study of women's experiences. Women Birth. 2016;29(2):189-95.

19. Keenan J, Stapleton H. Bonny babies? Motherhood and nurturing in the age of obesity. Health, Risk & Society. 2010;12(4):369-83.

20. Denison FC, Weir Z, Carver H, Norman JE, Reynolds RM. Physical activity in pregnant women with Class III obesity: A qualitative exploration of attitudes and behaviours. Midwifery. 2015;31(12):1163-7.

21. Nyman VM, Prebensen AK, Flensner GE. Obese women's experiences of encounters with midwives and physicians during pregnancy and childbirth. Midwifery. 2010;26(4):424-9.

22. Hastings-Tolsma M, Clark L, Nodine P, Teal S. Sterilization decision making among medically at-risk obese pregnant women. Qual Health Res. 2010;20(6):743-54.
