# Supplementary material for: Perceptions of risk and influences of choice in pregnant women with obesity. An evidence synthesis of qualitative research
Source: PLoS One. 2020 Jan 3;15(1):e0227325. doi: 10.1371/journal.pone.0227325 (PMC6941828; doi:10.1371/journal.pone.0227325)
Supplement: Attachment — Submitted filename: Response to reviewers.docx [file pone.0227325.s009.docx]

10^th^ Floor, North Wing

**Department of Women and Children’ Health**

St Thomas' Hospital

Westminster Bridge Road

London SE1 7EH

Office Direct Line: 020 7188 7804

Email: [sophie.relph@kcl.ac.uk](mailto:sophie.relph@kcl.ac.uk)

11^th^ December 2019

Dear Editor,

**Re: Revision required: PONE-D-19-31821 - EMID:1fed788ae74c03d7**

# Thank you for your review and comments on our paper entitled ‘Perceptions of risk and influences of choice in pregnant women with obesity. An Evidence Synthesis of Qualitative Research’. Please accept this resubmission of the paper, with response to comments summarised below.

Comments from Reviewer:

1. Use of the word 'influencers' feels a little awkward and does not add anything over 'influences' suggest change to influences.
   - Response: Thank you for this suggestion. We have changed the terminology used throughout to ensure it is consistent.
2. Check the acronym HCP is defined at first use.
   - Response: Thank you for pointing this out. Whilst the acronym has been defined in the text, it was not with the first reference to healthcare professionals. The reference has therefore been moved.
3. Line 97 sentence beginning 'In studies where women of weight in the non-obese range were included...' also feels a little awkward. I suggest the authors consider rewording.
   - Response: We have reworded as suggested. The sentence now reads: ‘*Only quotations or interpretations which were directly inferred from participants with obesity were synthesised from studies which included women of other weight ranges’.*

We look forward to your further review,

Yours sincerely,

Dr. Sophie Relph
